# Towards cascading genetic risk in Alzheimer’s disease

**DOI:** 10.1093/brain/awae176

**Published:** 2024-05-31

**Authors:** Andre Altmann, Leon M Aksman, Neil P Oxtoby, Alexandra L Young, ADNI, Michael Weiner, Paul Aisen, Ronald Petersen, Michael Weiner, Paul Aisen, Ronald Petersen, Clifford R Jack, William Jagust, Susan Landau, Monica Rivera-Mindt, Ozioma Okonkwo, Leslie M Shaw, Edward B Lee, Arthur W Toga, Laurel Beckett, Danielle Harvey, Robert C Green, Andrew J Saykin, Kwangsik Nho, Richard J Perrin, Duygu Tosun, Pallavi Sachdev, Robert C Green, Tom Montine, Cat Conti, Michael W Weiner, Rachel Nosheny, Juliet Fockler, Melanie J Miller, Catherine Conti, Winnie Kwang, Chengshi Jin, Adam Diaz, Miriam Ashford, Derek Flenniken, Adrienne Kormos, Ronald Petersen, Paul Aisen, Michael Rafii, Rema Raman, Gustavo Jimenez, Michael Donohue, Jennifer Salazar, Andrea Fidell, Virginia Boatwright, Justin Robison, Caileigh Zimmerman, Yuliana Cabrera, Sarah Walter, Taylor Clanton, Elizabeth Shaffer, Caitlin Webb, Lindsey Hergesheimer, Stephanie Smith, Sheila Ogwang, Olusegun Adegoke, Payam Mahboubi, Jeremy Pizzola, Cecily Jenkins, Laurel Beckett, Danielle Harvey, Michael Donohue, Naomi Saito, Adam Diaz, Kedir Adem Hussen, Ozioma Okonkwo, Monica Rivera-Mindt, Hannatu Amaza, Mai Seng Thao, Shaniya Parkins, Omobolanle Ayo, Matt Glittenberg, Isabella Hoang, Kaori Kubo Germano, Joe Strong, Trinity Weisensel, Fabiola Magana, Lisa Thomas, Vanessa Guzman, Adeyinka Ajayi, Joseph Di Benedetto, Sandra Talavera, Clifford R Jack, Joel Felmlee, Nick C Fox, Paul Thompson, Charles DeCarli, Arvin Forghanian-Arani, Bret Borowski, Calvin Reyes, Caitie Hedberg, Chad Ward, Christopher Schwarz, Denise Reyes, Jeff Gunter, John Moore-Weiss, Kejal Kantarci, Leonard Matoush, Matthew Senjem, Prashanthi Vemuri, Robert Reid, Ian Malone, Sophia I Thomopoulos, Talia M Nir, Neda Jahanshad, Alexander Knaack, Evan Fletcher, Danielle Harvey, Duygu Tosun-Turgut, Stephanie Rossi Chen, Mark Choe, Karen Crawford, Paul A Yushkevich, Sandhitsu Das, William Jagust, Susan Landau, Robert A Koeppe, Gil Rabinovici, Victor Villemagne, Brian LoPresti, Richard J Perrin, John Morris, Erin Franklin, Haley Bernhardt, Nigel J Cairns, Lisa Taylor-Reinwald, Leslie Shaw, Edward B Lee, M Y Virginia Lee, Magdalena Korecka, Magdalena Brylska, Yang Wan, J Q Trojanowki, Arthur W Toga, Karen Crawford, Scott Neu, Andrew J Saykin, Kwangsik Nho, Tatiana M Foroud, Taeho Jo, Shannon L Risacher, Hannah Craft, Liana G Apostolova, Kelly Nudelman, Kelley Faber, Zoë Potter, Kaci Lacy, Rima Kaddurah-Daouk, Li Shen, Jason Karlawish, Claire Erickson, Joshua Grill, Emily Largent, Kristin Harkins, Michael W Weiner, Leon Thal, Zaven Kachaturian, Richard Frank, Peter J Snyder, Neil Buckholtz, John K Hsiao, Laurie Ryan, Susan Molchan, Zaven Khachaturian, Maria Carrillo, William Potter, Lisa Barnes, Marie Bernard, Hector González, Carole Ho, John K Hsiao, Jonathan Jackson, Eliezer Masliah, Donna Masterman, Ozioma Okonkwo, Richard Perrin, Laurie Ryan, Nina Silverberg, Lisa Silbert, Jeffrey Kaye, Sylvia White, Aimee Pierce, Amy Thomas, Tera Clay, Daniel Schwartz, Gillian Devereux, Janet Taylor, Jennifer Ryan, Mike Nguyen, Madison DeCapo, Yanan Shang, Lon Schneider, Cynthia Munoz, Diana Ferman, Carlota Conant, Katherin Martin, Kristin Oleary, Sonia Pawluczyk, Elizabeth Trejo, Karen Dagerman, Liberty Teodoro, Mauricio Becerra, Madiha Fairooz, Sonia Garrison, Julia Boudreau, Yair Avila, James Brewer, Aaron Jacobson, Antonio Gama, Chi Kim, Emily Little, Jennifer Frascino, Nichol Ferng, Socorro Trujillo, Judith Heidebrink, Robert Koeppe, Steven MacDonald, Dariya Malyarenko, Jaimie Ziolkowski, James O'Connor, Nicole Robert, Suzan Lowe, Virginia Rogers, Ronald Petersen, Barbara Hackenmiller, Bradley Boeve, Colleen Albers, Connie Kreuger, David Jones, David Knopman, Hugo Botha, Jessica Magnuson, Jonathan Graff-Radford, Kerry Crawley, Michael Schumacher, Sanna McKinzie, Steven Smith, Tascha Helland, Val Lowe, Vijay Ramanan, Valory Pavlik, Jacob Faircloth, Jeffrey Bishop, Jessica Nath, Maria Chaudhary, Maria Kataki, Melissa Yu, Nathiel Pacini, Randall Barker, Regan Brooks, Ruchi Aggarwal, Lawrence Honig, Yaakov Stern, Akiva Mintz, Jonathan Cordona, Michelle Hernandez, Justin Long, Abbey Arnold, Alex Groves, Anna Middleton, Blake Vogler, Cierra McCurry, Connie Mayo, Cyrus Raji, Fatima S Amtashar, Heather Klemp, Heather Nicole Elmore, James Ruszkiewicz, Jasmina Kusuran, Jasmine Stewart, Jennifer Horenkamp, Julia Greeson, Kara Wever, Katie Vo, Kelly Larkin, Lesley Rao, Lisa Schoolcraft, Lora Gallagher, Madeline Paczynski, Maureen McMillan, Michael Holt, Nicole Gagliano, Rachel Henson, Renee LaBarge, Robert Swarm, Sarah Munie, Serena Cepeda, Stacey Winterton, Stephen Hegedus, TaNisha Wilson, Tanya Harte, Zach Bonacorsi, David Geldmacher, Amber Watkins, Brandi Barger, Bryan Smelser, Charna Bates, Cynthia Stover, Emily McKinley, Gregory Ikner, Haley Hendrix, Harold Matthew Cooper, Jennifer Mahaffey, Lindsey Booth Robbins, Loren Brown Ashley, Marissa Natelson-Love, Princess Carter, Veronika Solomon, Hillel Grossman, Alexandra Groome, Allison Ardolino, Anthony Kaplan, Faye Sheppard, Genesis Burgos-Rivera, Gina Garcia-Camilo, Joanne Lim, Judith Neugroschl, Kimberly Jackson, Kirsten Evans, Laili Soleimani, Mary Sano, Nasrin Ghesani, Sarah Binder, Xiomara Mendoza Apuango, Ajay Sood, Amelia Troutman, Kimberly Blanchard, Arlene Richards, Grace Nelson, Kirsten Hendrickson, Erin Yurko, Jamie Plenge, Victoria Rufo, Raj Shah, Ranjan Duara, Brendan Lynch, Cesar Chirinos, Christine Dittrich, Debbie Campbell, Diego Mejia, Gilberto Perez, Helena Colvee, Joanna Gonzalez, Josalen Gondrez, Joshua Knaack, Mara Acevedo, Maria Cereijo, Maria Greig-Custo, Michelle Villar, Morris Wishnia, Sheryl Detling, Warren Barker, Marilyn Albert, Abhay Moghekar, Barbara Rodzon, Corey Demsky, Gregory Pontone, Jim Pekar, Leonie Farrington, Martin Pomper, Nicole Johnson, Tolulope Alo, Martin Sadowski, Anaztasia Ulysse, Arjun Masurkar, Brittany Marti, David Mossa, Emilie Geesey, Emily Petrocca, Evan Schulze, Jennifer Wong, Joseph Boonsiri, Sunnie Kenowsky, Tatianne Martinez, Veronica Briglall, P Murali Doraiswamy, Adaora Nwosu, Alisa Adhikari, Cammie Hellegers, Jeffrey Petrella, Olga James, Terence Wong, Thomas Hawk, Sanjeev Vaishnavi, Hannah McCoubrey, Ilya Nasrallah, Rachel Rovere, Jeffrey Maneval, Elizabeth Robinson, Francisco Rivera, Jade Uffelman, Martha Combs, Patricia O'Donnell, Sara Manning, Richard King, Alayne Nieto, Amanda Glueck, Anjana Mandal, Audrie Swain, Bethanie Gamble, Beverly Meacham, Denece Forenback, Dorothy Ross, Elizabeth Cheatham, Ellen Hartman, Gary Cornell, Jordan Harp, Laura Ashe, Laura Goins, Linda Watts, Morgan Yazell, Prabin Mandal, Regan Buckler, Sylvia Vincent, Triana Rudd, Oscar Lopez, Ann Arlene Malia, Caitlin Chiado, Cary Zik, James Ruszkiewicz, Kathleen Savage, Linda Fenice, MaryAnn Oakley, Paige C Tacey, Sarah Berman, Sarah Bowser, Stephen Hegedus, Xanthia Saganis, Anton Porsteinsson, Abigail Mathewson, Asa Widman, Bridget Holvey, Emily Clark, Esmeralda Morales, Iris Young, James Ruszkiewicz, Kevin Hopkins, Kimberly Martin, Nancy Kowalski, Rebecca Hunt, Roberta Calzavara, Russell Kurvach, Stephen D'Ambrosio, Gaby Thai, Beatriz Vides, Brigit Lieb, Catherine McAdams-Ortiz, Cyndy Toso, Ivan Mares, Kathryn Moorlach, Luter Liu, Maria Corona, Mary Nguyen, Melanie Tallakson, Michelle McDonnell, Milagros Rangel, Neetha Basheer, Patricia Place, Romina Romero, Steven Tam, Trung Nguyen, Abey Thomas, Alexander Frolov, Alka Khera, Amy Browning, Brendan Kelley, Courtney Dawson, Dana Mathews, Elaine Most, Elizeva Phillips, Lynn Nguyen, Maribel Nunez, Matalin Miller, Matthew R Jones, Natalie Martinez, Rebecca Logan, Roderick McColl, Sari Pham, Tiffani Fox, Tracey Moore, Allan Levey, Abby Brown, Andrea Kippels, Ashton Ellison, Casie Lyons, Chadwick Hales, Cindy Parry, Courtney Williams, Elizabeth McCorkle, Guy Harris, Heather Rose, Inara Jooma, Jahmila Al-Amin, James Lah, James Webster, Jessica Swiniarski, Latasha Chapman, Laura Donnelly, Lauren Mariotti, Mary Locke, Phyllis Vaughn, Rachael Penn, Sallie Carpentier, Samira Yeboah, Sarah Basadre, Sarah Malakauskas, Stefka Lyron, Tara Villinger, Terra Burney, Jeffrey Burns, Ala Abusalim, Alexandra Dahlgren, Alexandria Montero, Anne Arthur, Heather Dooly, Katelynn Kreszyn, Katherine Berner, Lindsey Gillen, Maria Scanlan, Mercedes Madison, Nicole Mathis, Phyllis Switzer, Ryan Townley, Samantha Fikru, Samantha Sullivan, Ella Wright, Maryam Beigi, Anthony Daley, Ashley Ko, Brittney Luong, Glen Nyborg, Jessica Morales, Kelly Durbin, Lauren Garcia, Leila Parand, Lorena Macias, Lorena Monserratt, Maya Farchi, Pauline Wu, Robert Hernandez, Thao Rodriguez, Neill Graff-Radford, A'llana Marolt, Anton Thomas, Deborah Aloszka, Ercilia Moncayo, Erin Westerhold, Gregory Day, Kandise Chrestensen, Mary Imhansiemhonehi, Sanna McKinzie, Sochenda Stephens, Sylvia Grant, Jared Brosch, Amy Perkins, Aubree Saunders, Debra Silberberg Kovac, Heather Polson, Isabell Mwaura, Kassandra Mejia, Katherine Britt, Kathy King, Kayla Nichols, Kayley Lawrence, Lisa Rankin, Martin Farlow, Patricia Wiesenauer, Robert Bryant, Scott Herring, Sheryl Lynch, Skylar Wilson, Traci Day, William Korst, Christopher van Dyck, Adam Mecca, Alyssa Miller, Amanda Brennan, Amber Khan, Audrey Ruan, Carol Gunnoud, Chelsea Mendonca, Danielle Raynes-Goldfinger, Elaheh Salardini, Elisa Hidalgo, Emma Cooper, Erawadi Singh, Erin Murphy, Jeanine May, Jesse Stanhope, Jessica Lam, Julia Waszak, Kimberly Nelsen, Kimberly Sacaza, Mayer Joshua Hasbani, Meghan Donahue, Ming-Kai Chen, Nicole Barcelos, Paul Eigenberger, Robin Bonomi, Ryan O'Dell, Sarah Jefferson, Siddharth Khasnavis, Stephen Smilowitz, Susan DeStefano, Susan Good, Terry Camarro, Vanessa Clayton, Yanis Cavrel, YuQuan Oliver Lu, Howard Chertkow, Howard Bergman, Chris Hosein, Sandra Black, Anish Kapadia, Aparna Bhan, Benjamin Lam, Christopher Scott, Gillian Gabriel, Jennifer Bray, Ljubica Zotovic, Maria Samira Gutierrez, Mario Masellis, Marjan Farshadi, Maurylette Gui, Meghan Mitchell, Rebecca Taylor, Ruby Endre, Zhala Taghi-Zada, Robin Hsiung, Carolyn English, Ellen Kim, Eugene Yau, Haley Tong, Laura Barlow, Lauren Jennings, Michele Assaly, Paula Nunes, Tahlee Marian, Andrew Kertesz, John Rogers, Dick Trost, Dylan Wint, Charles Bernick, Donna Munic, Ian Grant, Aaliyah Korkoyah, Ali Raja, Allison Lapins, Caila Ryan, Jelena Pejic, Kailey Basham, Leena Lukose, Loreece Haddad, Lucas Quinlan, Nathaniel Houghtaling, Carl Sadowsky MD, Walter Martinez MD, Teresa Villena MD, Brigid Reynolds, Angelica Forero, Carolyn Ward, Emma Brennan, Esteban Figueroa, Giuseppe Esposito, Jessica Mallory, Kathleen Johnson, Kathryn Turner, Katie Seidenberg, Kelly McCann, Margaret Bassett, Melanie Chadwick, Raymond Scott Turner, Robin Bean, Saurabh Sharma, Gad Marshall, Aferdita Haviari, Alison Pietras, Bradley Wallace, Catherine Munro, Gladiliz Rivera-Delpin, Hadley Hustead, Isabella Levesque, Jennifer Ramirez, Karen Nolan, Kirsten Glennon, Mariana Palou, Michael Erkkinen, Nicole DaSilva, Pamela Friedman, Regina M Silver, Ricardo Salazar, Roxxanne Polleys, Scott McGinnis, Seth Gale, Tia Hall, Tuan Luu, Steven Chao, Emmeline Lin, Jaila Coleman, Kevin Epperson, Minal Vasanawala, Alireza Atri, Amy Rangel, Brittani Evans, Candy Monarrez, Carol Cline, Carolyn Liebsack, Daniel Bandy, Danielle Goldfarb, Debbie Intorcia, Jennifer Olgin, Kelly Clark, Kelsey King, Kylee York, Marina Reade, Michael Callan, Michael Glass, Michaela Johnson, Michele Gutierrez, Molly Goddard, Nadira Trncic, Parichita Choudhury, Priscilla Reyes, Serena Lowery, Shaundra Hall, Sonia Olgin, Stephanie de Santiago, Michael Alosco, Alyssa Ton, Amanda Jimenez, Andrew Ellison, Anh Tran, Brandon Anderson, Della Carter, Donna Veronelli, Steven Lenio, Eric Steinberg, Jesse Mez, Jason Weller, Jennifer Johns, Jesse Mez, Jessica Harkins, Alexa Puleio, Ina Hoti, Jane Mwicigi, Alexa Puleio, Michael Alosco, Olivia Schultz, Mona Lauture, Eric Steinberg, Ridiane Denis, Ronald Killiany, Sarab Singh, Steven Lenio, Wendy Qiu, Ycar Devis, Thomas Obisesan, Andrew Stone, Debra Ordor, Ifreke Udodong, Immaculata Okonkwo, Javed Khan, Jillian Turner, Kyliah Hughes, Oshoze Kadiri, Charles Duffy, Ariana Moss, Katherine Stapleton, Maria Toth, Marianne Sanders, Martin Ayres, Melissa Hamski, Parianne Fatica, Paula Ogrocki, Sarah Ash, Stacy Pot, Doris Chen, Andres Soto, Costin Tanase, David Bissig, Hafsanoor Vanya, Heather Russell, Hitesh Patel, Hongzheng Zhang, Kelly Wallace, Kristi Ayers, Maria Gallegos, Martha Forloines, Meghan Sinn, Queennie Majorie S Kahulugan, Richard Isip, Sandra Calderon, Talia Hamm, Michael Borrie, T-Y Lee, Rob Bartha, Sterling Johnson, Sanjay Asthana, Cynthia M Carlsson, Allison Perrin, Pierre Tariot, Adam Fleisher, Stephanie Reeder, Horacio Capote, Allison Emborsky, Anna Mattle, Bela Ajtai, Benjamin Wagner, Bennett Myers, Daryn Slazyk, Delaney Fragale, Erin Fransen, Heather Macnamara, Jonathan Falletta, Joseph Hirtreiter, Laszlo Mechtler, Megan King, Michael Asbach, Michelle Rainka, Richard Zawislak, Scott Wisniewski, Stephanie O'Malley, Tatiana Jimenez-Knight, Todd Peehler, Traci Aladeen, Vernice Bates, Violet Wenner, Wisam Elmalik, Douglas W Scharre, Arun Ramamurthy, Soumya Bouchachi, Maria Kataki, Rawan Tarawneh, Brendan Kelley, Dzintra Celmins, Alicia Leader, Chris Figueroa, Heather Bauerle, Katlynn Patterson, Michael Reposa, Steven Presto, Tuba Ahmed, Wendy Stewart, Godfrey D Pearlson, Karen Blank, Karen Anderson, Robert B Santulli, Eben S Schwartz, Jeff Williamson, Alicia Jessup, Andrea Williams, Crystal Duncan, Abigail O'Connell, Karen Gagnon, Ezequiel Zamora, James Bateman, Freda Crawford, Deb Thompson, Eboni Walker, Jennifer Rowell, Mikell White, Phillip Hunter Ledford, Sarah Bohlman, Susan Henkle, Joseph Bottoms, Lena Moretz, Bevan Hoover, Michael Shannon, Samantha Rogers, Wendy Baker, William Harrison, Chuang-Kuo Wu, Alexis DeMarco, Ava Stipanovich, Daniel Arcuri, Jan Clark, Jennifer Davis, Kerstin Doyon, Marie Amoyaw, Mauro Veras Acosta, Ronald Bailey, Scott Warren, Terry Fogerty, Victoria Sanborn, Butler Hospital, Meghan Riddle, Stephen Salloway, Paul Malloy, Stephen Correia, Charles Windon, Morgan Blackburn, Howard J Rosen, Bruce L Miller, Amanda Smith, Ijeoma Mba, Jenny Echevarria, Juris Janavs, Emily Roglaski, Meagan Yong, Rebecca Devine, Hamid Okhravi, Edgardo Rivera, Teresa Kalowsky, Caroline Smith, Christina Rosario, Joseph Masdeu, Richard Le, Maushami Gurung, Marwan Sabbagh, Angelica Garcia, Micah Ellis Slaughter, Nadeen Elayan, Skieff Acothley, Nunzio Pomara, Raymundo Hernando, Vita Pomara, Chelsea Reichert, Olga Brawman-Mintzer, Allison Acree, Arthur Williams, Campbell Long, Rebecca Long, Paul Newhouse, Sydni Jenee Hill, Amy Boegel, Sudha Seshadri, Amy Saklad, Floyd Jones, William Hu, V Sotelo, Yaneicy Gonazalez Rojas, Jacobo Mintzer, Crystal Flynn Longmire, Kenneth Spicer, Daniel C Alexander, Frederik Barkhof, Maryam Shoai, John Hardy, Jonathan M Schott

**Affiliations:** UCL Centre for Medical Image Computing, Department of Medical Physics and Biomedical Engineering, University College London, London, WC1E 6BT, UK; Stevens Neuroimaging and Informatics Institute, Keck School of Medicine, University of Southern California, Los Angeles, CA 90033, USA; UCL Centre for Medical Image Computing, Department of Computer Science, University College London, London, WC1E 6BT, UK; UCL Centre for Medical Image Computing, Department of Computer Science, University College London, London, WC1E 6BT, UK; UCL Centre for Medical Image Computing, Department of Computer Science, University College London, London, WC1E 6BT, UK; UCL Centre for Medical Image Computing, Department of Medical Physics and Biomedical Engineering, University College London, London, WC1E 6BT, UK; UCL Queen Square Institute of Neurology, University College London, London, WC1N 3BG, UK; Department of Radiology and Nuclear Medicine, Amsterdam University Medical Center, Amsterdam, 1081 HV, The Netherlands; UCL Queen Square Institute of Neurology, University College London, London, WC1N 3BG, UK; UK Dementia Research Institute, University College London, London, WC1E 6BT, UK; UCL Queen Square Institute of Neurology, University College London, London, WC1N 3BG, UK; UK Dementia Research Institute, University College London, London, WC1E 6BT, UK; UK Dementia Research Institute, University College London, London, WC1E 6BT, UK; Dementia Research Centre, UCL Queen Square Institute of Neurology, University College London, London, WC1N 3AR, UK

**Keywords:** Alzheimer’s disease, *APOE*, polygenic risk, longitudinal progression, biomarker

## Abstract

Alzheimer’s disease typically progresses in stages, which have been defined by the presence of disease-specific biomarkers: amyloid (A), tau (T) and neurodegeneration (N). This progression of biomarkers has been condensed into the ATN framework, in which each of the biomarkers can be either positive (+) or negative (−). Over the past decades, genome-wide association studies have implicated ∼90 different loci involved with the development of late-onset Alzheimer’s disease. Here, we investigate whether genetic risk for Alzheimer’s disease contributes equally to the progression in different disease stages or whether it exhibits a stage-dependent effect.

Amyloid (A) and tau (T) status was defined using a combination of available PET and CSF biomarkers in the Alzheimer’s Disease Neuroimaging Initiative cohort. In 312 participants with biomarker-confirmed A−T− status, we used Cox proportional hazards models to estimate the contribution of *APOE* and polygenic risk scores (beyond *APOE*) to convert to A+T− status (65 conversions). Furthermore, we repeated the analysis in 290 participants with A+T− status and investigated the genetic contribution to conversion to A+T+ (45 conversions). Both survival analyses were adjusted for age, sex and years of education.

For progression from A−T− to A+T−, APOE-e4 burden showed a significant effect [hazard ratio (HR) = 2.88; 95% confidence interval (CI): 1.70–4.89; *P* < 0.001], whereas polygenic risk did not (HR = 1.09; 95% CI: 0.84–1.42; *P* = 0.53). Conversely, for the transition from A+T− to A+T+, the contribution of APOE-e4 burden was reduced (HR = 1.62; 95% CI: 1.05–2.51; *P* = 0.031), whereas the polygenic risk showed an increased contribution (HR = 1.73; 95% CI: 1.27–2.36; *P* < 0.001). The marginal *APOE* effect was driven by e4 homozygotes (HR = 2.58; 95% CI: 1.05–6.35; *P* = 0.039) as opposed to e4 heterozygotes (HR = 1.74; 95% CI: 0.87–3.49; *P* = 0.12).

The genetic risk for late-onset Alzheimer’s disease unfolds in a disease stage-dependent fashion. A better understanding of the interplay between disease stage and genetic risk can lead to a more mechanistic understanding of the transition between ATN stages and a better understanding of the molecular processes leading to Alzheimer’s disease, in addition to opening therapeutic windows for targeted interventions.

## Teaser

Alzheimer’s disease typically progresses in stages defined by the presence of amyloid (A), tau (T), and neurodegeneration (N). Altmann *et al.* use survival analysis to examine whether genetic risk for Alzheimer’s disease contributes equally to transition between all ATN stages, or whether it instead exhibits stage-dependent effects.

**See M-Carlgren (https://doi.org/10.1093/brain/awae237) for a scientific commentary on this article.**

## Introduction

Alzheimer’s disease is characterized, at the neuropathological level, by the build-up of two proteins: amyloid plaques and neurofibrillary tangles of phosphorylated tau.^1^ Both these pathological features can be observed long before the memory loss and decline in executive function that is characteristic of patients with Alzheimer’s disease. Accumulation of amyloid plaques in the brain predates the clinical symptoms of Alzheimer’s disease by two decades,^2^ whereas the spatial distribution of tau tangles reflects more closely the reported cognitive deficits and neurodegeneration.^3^

The amyloid cascade hypothesis postulates that the deposition of the amyloid-β protein (the main component of the amyloid plaques) is the cause of Alzheimer’s disease and that neurofibrillary tangles, cell loss, vascular damage and dementia are a direct consequence.^4^ In keeping with the amyloid cascade hypothesis, a theoretical framework for the progression of biomarkers during the course of Alzheimer’s disease has been developed.^5^ Here, amyloid pathology is the first to appear, followed by tau pathology, neurodegeneration and, finally, cognitive decline. Support for this theoretical framework comes from a number of lines of evidence, including a variety of data-driven modelling approaches based on biomarker data.^6-10^ In an attempt to operationalize this, a simplified ATN model has been proposed.^11^ The components of the ATN model refer to the status of three different key biomarkers in Alzheimer’s disease: amyloid (A), tau (T) and neurodegeneration (N). In this approach, each of the three biomarkers can be either positive or negative. Exceeding the centiloid threshold in amyloid PET imaging would place a participant into the A-positive (A+) group, whereas a scan slightly below the threshold would be considered amyloid negative (A−). One practical advantage (but also a major source of criticism) of this model is that the biomarker status can be assessed using a variety of methods: wet biomarkers (CSF or plasma) or brain imaging (centiloids or visual reads).^12^ Although the progression from A−T−N− to A+T−N− to A+T+N− to A+T+N+ would be the most typical progression in Alzheimer’s disease and in keeping with the theoretical framework of biomarker progression, all combinations of biomarker statuses emerge in observational cohorts.^13,14^

Genome-wide association studies (GWASs) have broadened the understanding of the genetic basis of late-onset Alzheimer’s disease over the last decades.^15^ Initially, these studies were restricted to cases with a clinical diagnosis of Alzheimer’s disease and healthy controls.^16-18^ Recently, these GWASs have been enriched with participants with a family history of Alzheimer’s disease (diagnosis-by-proxy),^19-21^ leading to a drastic increase in sample size and expanding the set of genetic risk loci for Alzheimer’s disease to 90.^15^ These loci have been linked to various molecular processes, such as immunity, cholesterol processing and endocytosis.^22^ Further studies investigated the genetic effects on Alzheimer’s disease-related biomarkers, ranging from tau and amyloid levels in CSF^23,24^ or in the brain^25,26^ to MRI-based measures, such as hippocampal volume^27^ and phenotypes derived from disease progression modelling.^28^ The strongest common genetic risk factor for Alzheimer’s disease is the e4 allele of the *APOE* gene: carriers of the e4 allele have a 2- to 4-fold increased risk of developing Alzheimer’s disease, and e4 homozygous subjects have an 8- to 12-fold increased risk.^29^ The genetic risk outside the *APOE* region is often summarized using polygenic scores, which have been shown to improve predictions of clinical diagnosis^30^ and pathology-confirmed cases.^31^ Likewise, the effect of *APOE* and the polygenic risk on various imaging and non-imaging biomarkers have been investigated,^32-35^ with ongoing work suggesting that risk accumulated along different molecular pathways exerts differential effects on different biomarkers in Alzheimer’s disease.^35-37^

For Alzheimer’s disease and for other disorders, genetic risk is often considered as a time-invariant constant. That is, genetic risk identified through case–control studies is assumed to affect both onset and progression. However, given that Alzheimer’s disease is now understood to unfold in stages, we hypothesized, in line with the amyloid cascade hypothesis and the A/T/N framework, that genetic risk in Alzheimer’s disease is disease-stage dependent; i.e. some genetic risk factors will aid the transition from A− to A+, whereas other, distinct genetic risk factors will increase the risk to transition from T− to T+.

In this work, we explore whether genetic vulnerability to Alzheimer’s disease varies with disease stage. Using longitudinal data from the Alzheimer’s Disease Neuroimaging Imitative (ADNI) and survival analysis, we show that *APOE* contributes to progression from A−T− to A+T−, but only marginally from A+T− to A+T+. Conversely, polygenic risk contributes to the progression from A+T− to A+T+, but not from A−T− to A+T−.

## Materials and methods

### Data

Data used in the preparation of this article were obtained from the ADNI database (http://adni.loni.usc.edu). The ADNI was launched in 2003 as a public–private partnership, led by Principal Investigator Michael W. Weiner, MD. The primary goal of the ADNI has been to test whether serial MRI, PET, other biological markers, and clinical and neuropsychological assessment can be combined to measure the progression of mild cognitive impairment and early Alzheimer’s disease. For up-to-date information, see https://adni.loni.usc.edu/about/. ADNI study data were accessed through the R package ADNIMERGE (accessed: 20 July 2023).

### Preparation of genetic data

The genetic data preparation followed the procedure described by Altmann *et al*.^32^ The additional genetic data contributed by the ADNI-3 cohort was integrated with the existing data using the same processing pipeline. Briefly, single nucleotide polymorphism (SNP) genotyping data were available for *n* = 2001 subjects across ADNI phases 1, 2, GO and 3. Genotyping was conducted using four different platforms: Human610-Quad, HumanOmniExpress, Omni 2.5 M and Illumina Infinium Global Screening Array v.2 (Illumina).^38^ Prior to imputation, we applied subject-level quality control (QC) steps based on call rate (10% cut-off) and concordance between chip-inferred sex and self-reported sex separately for the four genotyping arrays; all subjects were retained. At the SNP level, we conducted standard QC steps ensuring compatibility with the reference panel used for imputation [strand consistency, allele names, position, reference/alternative allele assignments and minor allele frequency discrepancy (0.2 cut-off)]. Imputation was carried out using the Sanger Imputation Server (https://imputation.sanger.ac.uk/), with SHAPEIT for phasing,^39^ positional Burrows–Wheeler transform^40^ for imputation and the entire Haplotype Reference Consortium (release 1.1) reference panel.^41^ Data from the four different genotyping platforms were imputed separately. As part of post-imputation QC, multi-allelic variants and SNPs with imputation INFO score of <0.3 were removed, and genotype calls with a posterior probability of <0.9 were set to missing (i.e. hard called). Following the initial QC, genotypes from the four platforms were merged. Additional information on the imputation and QC process is detailed by Scelsi *et al.*^28,42^ Using the merged data, we retained SNPs with minor allele frequency ≥ 1% and genotyping rate of >0.9.

SNPweights^43^ was used to infer genetic ancestry from genotyped SNPs. The reference panel comprised Central European, Yoruba Africans and East Asian samples from HapMap 3^44^ and native Americans from Reich *et al*.^45^ For this study, only participants with predicted central European ancestry of ≥80% were retained (*n* = 1851). Next, using the imputed and merged data genetic relatedness between central European participants was computed. Initially, the SNP content was restricted to SNPs with minor allele frequency ≥ 5%, and linkage disequilibrium (LD) pruning was carried out in PLINK v.1.9 (–indep-pairwise 1000 50 0.1). The genetic relatedness matrix was computed using the remaining autosomal SNPs, and the dataset was trimmed to remove subects with relatedness of >0.1 (–rel-cutoff 0.1), leading to *n* = 1833 unrelated participants.

### Definition of genetic risk

In this study, we focused on two sources of genetic risk: (i) the risk conferred through the *APOE* gene based on the genetic markers for APOE-e2 and APOE-e4; and (ii) the polygenic risk conferred by the remaining genome. As described previously,^32^ polygenic risk scores (PRSs) were computed using the software PRSice v.2.1.9.^46^ As base GWAS, the stage 1 results of the Alzheimer’s disease GWAS featuring a clinically defined Alzheimer’s disease phenotype was used.^18^ For PRS computation, SNPs with minor allele frequency ≥ 5% were considered, and SNPs were selected using LD clumping (1000 kb, *R*^2^ of 0.1 and *P*-value threshold of 1.0) within the ADNI cohort, missing SNPs were simply ignored at the subject level (using the setting –missing SET_ZERO), and the *APOE* region was excluded (hg19 coordinates chr19 from 44 400 000 to 46 500 000). For this study, we used only the *P*-value cut-off of 1.0 × 10^−8^ to build the PRS (Supplementary Table 1). PRSs were computed for all ADNI participants with genome-wide genotyping data. Of the remaining subjects, *n* = 417 ADNI-1 participants who contributed to the Alzheimer’s disease GWAS^18^ were excluded from the analysis to ensure independence between training and application dataset for PRS. Thus, *n* = 1416 unrelated participants with central European ancestry were eligible for inclusion in the study.

### Definition of amyloid status

For this project we relied on two modalities to define amyloid status: amyloid-β PET using the ^18^F-florbetapir and ^18^F-florbetaben PET tracers, and CSF measures of amyloid-β(1–42) using the Roche Elecsys® immunoassay. We used data processed by ADNI for both modalities. Detailed information on the PET processing is available elsewhere (https://adni.loni.usc.edu/methods/pet–analysis)^47^; and information on CSF amyloid-β(1–42) processing is detailed by Bittner *et al.*^48^ and Hansson *et al*.^49^ For CSF amyloid-β(1–42) we used the cut-off of 880 pg/ml,^49^ and for amyloid-β PET we used the tracer-specific standardized uptake value ratio (SUVR) (whole cerebellum reference) cut-offs of 1.11 and 1.08 for ^18^F-florbetapir and the ^18^F-florbetaben,^50^ respectively.

A participant’s visit was labelled as A+ if either the PET result or the CSF result indicated a positive amyloid finding (i.e. in cases where the results were discordant, the visit would be labelled as A+). Visits with only negative amyloid findings were labelled as A−, and visits without any information on amyloid (i.e. neither a PET nor a CSF result) were labelled as ‘amyloid missing’.

### Definition of tau status

In keeping with our definition of amyloid positivity, we used available data from CSF and PET imaging to define the tau status. More precisely, we used the Phospho-Tau(^181^P) CSF Roche Elecsys® immunoassay and PET imaging using the ^18^F-flortaucipir tracer. Details on the processing are available elsewhere (https://adni.loni.usc.edu/methods/pet–analysis).^48,49,51^ For CSF we used phosphorylated ^181^P tau (pTau) with a cut-off of 34.61 pg/ml, and for tau PET we used the cut-off of 1.42 in the meta temporal region of interest comprising the amygdala, entorhinal cortex, fusiform gyrus, inferior and middle temporal gyri^52^ when normalized to the inferior cerebellar grey matter.^53^ Both cut-offs were data driven: (i) the tau PET cut-off corresponds to a *z*-score of 2.0 in the cognitively normal participants in ADNI (*n* = 506) and is close to the ‘high tau’ cut-off of 1.43 defined by Jack Jr *et al*.^54^; and (ii) the pTau cut-off was set to maximize the Youden’s index between CSF pTau and tau PET (at the 1.42 cut-off) in (*n* = 502) ADNI participants with concurrent CSF and PET measurements. The same labelling scheme as for amyloid was applied: a visit was labelled as T+ if either the CSF or the PET indicated a positive finding, T− if there were only negative tau findings, and ‘tau missing’ if neither data on CSF pTau nor on tau PET were available.

### Statistical modelling

We used Cox proportional hazards models to investigate the genetic contribution of progressing (i) from A−T− to A+T−; and (ii) from A+T− to A+T+. For (i), we included every eligible participant with genetic data and who was A−T− based on their biomarker results as described above. This earliest A−T− visit was considered the ‘start’ visit (i.e. the status of previous visits was ignored). A subject was considered a converter when the available biomarkers indicated A+T− at a later visit. The time of the first A+T− biomarker finding after their initial A−T− visit was used as the conversion time. For non-converters, we recorded the last visit where both amyloid and tau biomarker information was available to define the maximal follow-up time. Likewise, for (ii) we included every participant with genetic data and who was A+T−. This first A+T− visit was considered the ‘start’ visit. A subject was considered a converter when the available biomarkers indicated A+T+ at a later visit. The time of the first A+T+ biomarker finding after their initial A+T− visit was used as the conversion time. As before, for non-converters we recorded the last visit when amyloid and tau biomarker information was available as their last point of contact.

For the analyses, the Cox proportional hazards model included age at the inclusion visit (i.e. either the A−T− or the A+T− visit), sex and education. As variables of interest, we also included genetic variables for PRS, in addition to allele counts of APOE-e2 and APOE-e4. The proportional hazards assumption was tested for each covariate and for the global model using statistical tests and graphical diagnostics based on scaled Schoenfeld residuals. As a measure of overall model performance, the concordance index (C-index) was computed for the following: (i) full models; (ii) models without *APOE*; (iii) models without PRS; and (iv) models without any genetics. Statistical tests were carried out in R (v.4.1.0) using the survival (v.3.5.5), survminer (v.0.4.9) and rms (v.6.8.0) packages.

### Sensitivity analyses

In addition to the two main analyses, we conducted a series of sensitivity analyses addressing the conversion definition, biomarker cut-offs, biomarker source and polygenic score source.

#### Conversion definition

To maximize the available data, we relaxed the requirement for both amyloid and tau biomarkers to be available at the same visit to define conversion. As before, both biomarkers at the same time were required to define the ‘start’ visit as either A−T− or A+T−. However, for defining progression from A−T− or A+T−, a single A+ or T+ visit was sufficient, respectively.

#### Biomarker cut-offs

We varied the tau PET cut-off from 1.0 to 3.0 standard deviations (SDs) above the mean in the cognitively normal ADNI participants. Notably, the lowest cut-off resulted in 1.31 and was close to the neuropathologically defined cut-off of 1.29 by Lowe *et al*.^55^ The pTau cut-offs were adjusted accordingly to maximize Youden’s index between CSF pTau and tau PET.

#### Biomarker source

To maximize data and follow-up time, the main analyses combined data from two biomarker sources: CSF and PET. Additional sensitivity analyses relied exclusively on either CSF biomarkers or PET biomarkers. For this analysis, pTau cut-offs were varied in the range from 22 to 31, covering the values of 24.25 and 29.19, which were found to indicate tau PET positivity in Braak III/IV and Braak V/VI regions, respectively.^56^ Tau PET cut-offs were varied, as before, from 1.0 to 3.0 SD above the mean in cognitively normal participants (i.e. in the same range as the main analysis).

#### Polygenic score source

To include more SNPs in the PRS, we explored the summary statistics on Alzheimer’s disease and related dementias by Bellenguez *et al*.^21^ We followed the same PRS pipeline as above and applied a *P*-value threshold of 5.0 × 10^−8^ (i.e. genome-wide significant), leading to 77 included SNPs (Supplementary Table 2).

## Results

Out of 16 401 visits recorded in ADNI, 3789 (23.1%) visits had both amyloid and tau biomarker data available. Concordance between PET- and CSF-based assessment was 80% and 81% for amyloid and tau, respectively. For both survival models, we identified ∼300 subjects in the ADNI database with both biomarkers available (Table 1): 312 individuals were A−T−, of whom 65 converted to A+T−. The mean age of the A−T− group was 71.3 (6.65) years, and there was an almost equal number of males and females (49.4% females). Two hundred and ninety individuals were A+T− at any stage, of whom 45 converted to A+T+. The mean age of the A+T− group was 73.2 (6.9) years, significantly older than the A−T− group (Student’s unpaired *t*-test: *t* = 3.50, *P* < 0.001). The fraction of females in that cohort was lower compared with the A−T− group (43.8% females), but not at a significant level (χ^2^ test; χ^2^ = 1.65; d.f. = 1; *P* = 0.19). The distribution of APOE-e4 alleles (χ^2^ test; χ^2^ = 81.1; d.f. = 2; *P* = 2.4 × 10^−18^) and APOE-e2 alleles (Fisher’s exact test; *P* = 0.0004) differed significantly between A−T− and A+T−. There were more APOE-e2 carriers and fewer APOE-e4 carriers in the A−T− group than in the A+T− group. The PRS did not differ between the A−T− and A+T− groups (Student’s two-tailed *t*-test; *t* = 0.84; d.f. = 600; *P* = 0.39). The cohort with the relaxed conversion criterion showed comparable characteristics (Supplementary Table 3).

**Table 1:** Demographics

Characteristic	A−T−			A+T−		
	Total	Stable	Converter	Total	Stable	Converter
*n*	312	247	65	290	245	45
Age, mean (SD), years	71.3 (6.65)	71.0 (6.47)	72.2 (7.25)	73.2 (6.9)	73.2 (6.9)	73.1 (6.8)
Sex (% female)	49.4	48.6	52.3	43.8	43.3	46.7
Diagnosis (CN/MCI/AD)	195/113/4	150/94/3	45/19/1	118/153/19	100/128/17	18/25/2
Education, mean (SD), years	16.7 (2.5)	16.6 (2.6)	17.3 (2.2)	16.4 (2.6)	16.6 (2.7)	15.5 (2.4)
Follow-up, mean (SD), years	5.0 (3.1)	4.7 (3.0)	6.4 (3.4)	4.1 (2.6)	3.8 (2.6)	5.6 (2.3)
Time to event, mean (SD), years	n/a	n/a	4.5 (2.7)	n/a	n/a	4.4 (2.5)
APOE-e4 (0/1/2)	252/57/3	207/40/0	45/17/3	137/119/34	121/99/25	16/20/9
APOE-e2 (0/1/2)	265/46/1	205/41/1	60/5/0	273/17/0	230/15/0	43/2/0
PRS, mean (SD)	0.013 (0.012)	0.013 (0.012)	0.013 (0.011)	0.013 (0.013)	0.012 (0.013)	0.020 (0.011)

AD = Alzheimer’s disease; CN = cognitively normal; MCI = mild cognitive impairment; PRS = polygenic risk score.

### APOE-e4 influences progression from A−T− to A+T−

**Figure 1:**
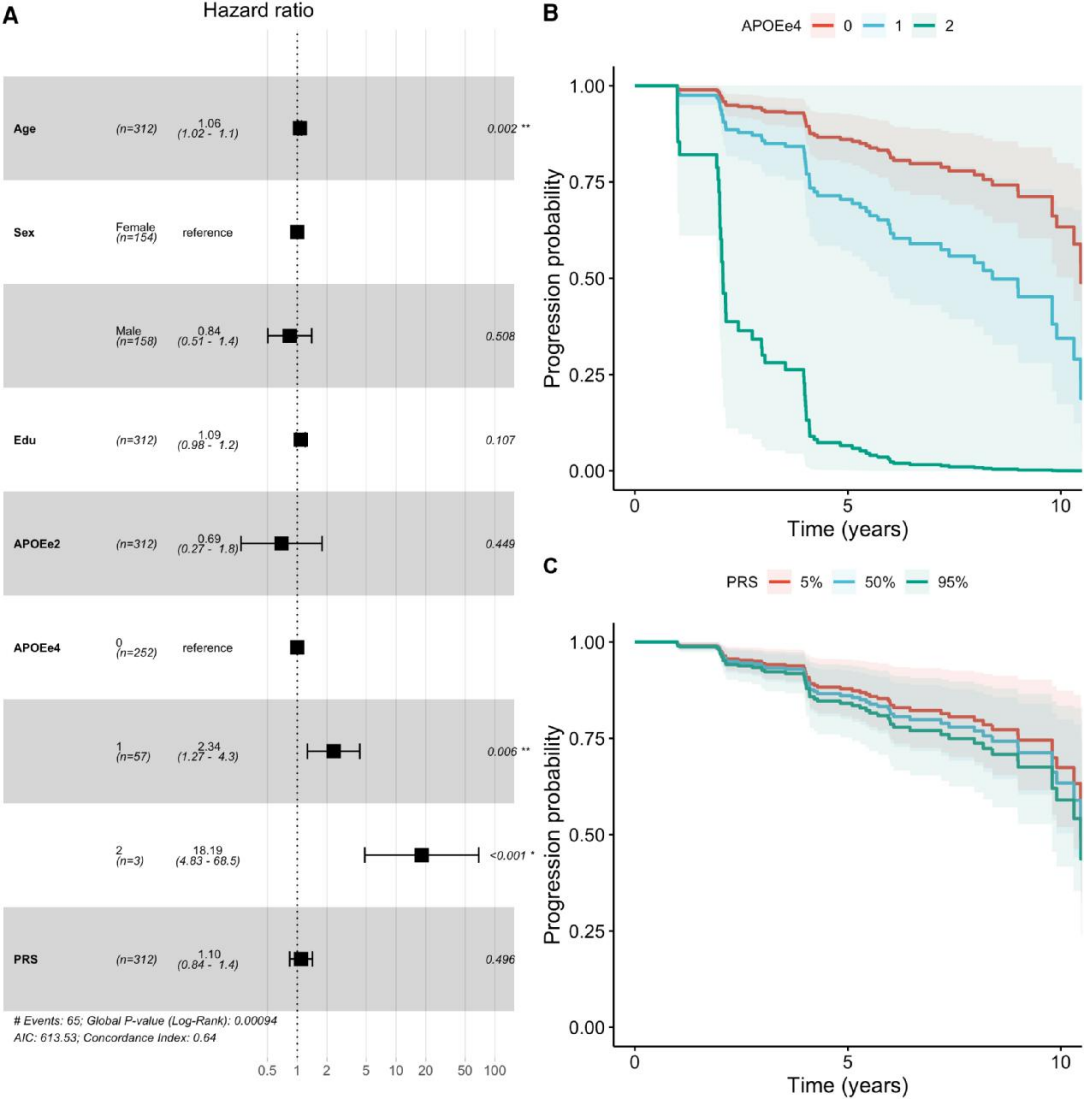
**Hazard ratios for the conversion from A−T− to A+T−**. (**A**) Forest plot depicting the HRs for all covariates in the model. (**B**) Estimated survival curves stratified by APOE-e4 genotype. (**C**) Estimated survival curves stratified by PRS percentile (5%, 50% and 95%). APOEe2 = number of APOE-e2 alleles; APOEe4 = number of APOE-e4 alleles; Edu = years of education; HR = hazard ratio; PRS = polygenic risk score, scaled to zero mean and unit standard deviation.

The survival analysis showed a significant contribution by APOE-e4 allele count [hazard ratio (HR) = 2.88; 95% confidence interval (CI): 1.70–4.89; *P* = 8.7 × 10^−5^] but no significant contribution by PRS (HR = 1.09; 95% CI: 0.84–1.42; *P* = 0.53) (Fig. 1). The APOE-e2 allele count directionally favoured a protective effect, but this was not significant (HR = 0.73; 95% CI: 0.28–1.86; *P* = 0.51). The Cox proportional hazards assumption held for this model (global Schoenfeld test, *P* = 0.24) (Supplementary Fig. 1). The C-indices aligned with this pattern: full model (C = 0.612), no *APOE* (C = 0.525), no PRS (C = 0.611) and no genetics (C = 0.531). This pattern of associations of *APOE* and PRS with progression was largely independent of the tau PET and pTau thresholds (Supplementary Table 4). Furthermore, using the more relaxed conversion criteria (i.e. confirmed A+ status was sufficient instead of a confirmation of A+T−) yielded more conversions (85 instead of 65), but qualitatively the same result (Supplementary Fig. 2), i.e. a significant contribution by APOE-e4 allele count (HR = 3.34; 95% CI: 2.14–5.22; *P* = 1.0 × 10^−7^) but not by PRS (HR = 1.06; 95% CI: 0.84–1.34; *P* = 0.61).

### Polygenic risk affects the progression from A+T− to A+T+

**Figure 2:**
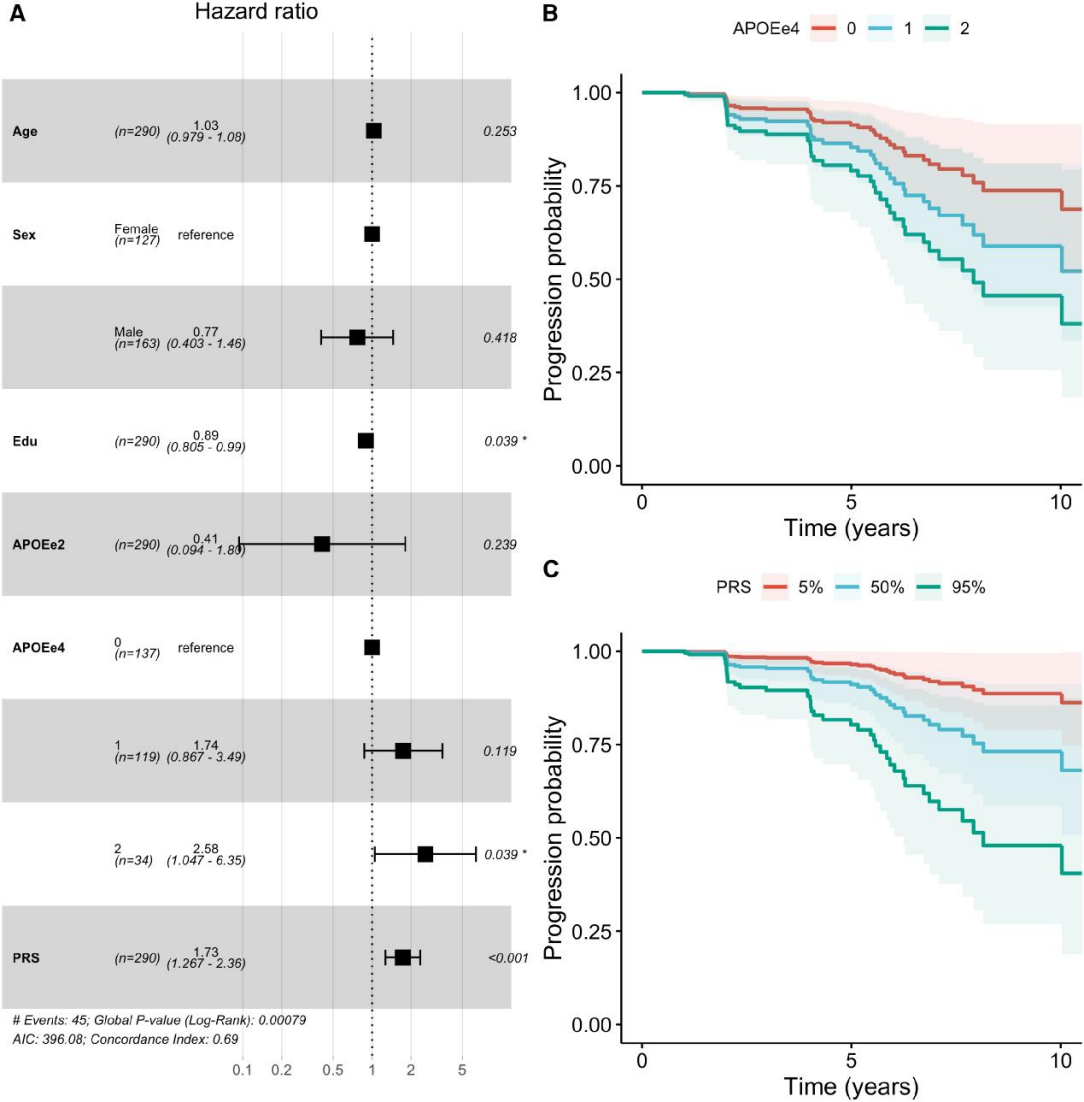
**Hazard ratios for the conversion from A+T− to A+T+**. (**A**) Forest plot depicting the hazard ratios for all covariates in the model. (**B**) Estimated survival curves stratified by APOE-e4 genotype. (**C**) Estimated survival curves stratified by PRS percentile (5%, 50% and 95%). APOEe2 = number of APOE-e2 alleles; APOEe4 = number of APOE-e4 alleles; Edu = years of education; PRS = polygenic risk score, scaled to zero mean and unit standard deviation.

The survival analysis showed a marginally significant contribution by APOE-e4 burden (HR = 1.62; 95% CI: 1.05–2.51; *P* = 0.031), which was mainly driven by APOE-e4 homozygotes (HR = 2.58; 95% CI: 1.05–6.35; *P* = 0.039) rather than APOE-e4 heterozygotes (HR = 1.74; 95% CI: 0.87–3.49; *P* = 0.12) (Fig. 2). Furthermore, there was a significant contribution by PRS (HR = 1.72; 95% CI: 1.27–2.36; *P* = 0.00057). The APOE-e2 allele count again was directionally consistent with a protective effect, but this was not significant (HR = 0.41; 95% CI: 0.09–1.78; *P* = 0.23). The Cox proportional hazards assumption held for this model (Supplementary Fig. 3). The C-indices dropped marginally when either *APOE* or PRS was removed from the model: full model (C = 0.657), no *APOE* (C = 0.634), no PRS (C = 0.615) and no genetics (C = 0.549). Moreover, the association pattern of *APOE* and PRS with progression was largely independent of the tau PET and pTau cut-offs (Supplementary Table 4). In addition, education showed a marginally protective association with conversion to A+T+ (HR = 0.89; 95% CI: 0.80–0.99; *P* = 0.039). Applying the more relaxed conversion criteria (i.e. confirmed T+ status was sufficient instead of a confirmation of A+T+) yielded more conversions (51 instead of 45), but qualitatively the same result (Supplementary Fig. 4): a significant contribution by PRS (HR = 1.62; 95% CI: 1.22–2.17; *P* = 0.001) and a marginal contribution by APOE-e4 allele burden (HR = 1.56; 95% CI: 1.04–2.35; *P* = 0.031).

### Results are independent of PRS source

Using an the alternative PRS with 77 SNPs led to the same observation of a significant effect by APOE-e4 burden on A−T− to A+T− conversion (HR = 2.84; 95% CI: 1.68–4.82; *P* = 0.0001) and a lack of contribution by the PRS (HR = 0.97; 95% CI: 0.77–1.2; *P* = 0.80; Supplementary Fig. 5A). Conversely, for A+T− to A+T+ conversion, the contribution by APOE-e4 was reduced (HR = 1.71; 95% CI: 1.09–2.68; *P* = 0.019), whereas the PRS exhibited a strong contribution (HR = 1.59; 95% CI: 1.19–2.13; *P* = 0.00163; Supplementary Fig. 5B).

### Results are independent of biomarker source

**Figure 3:**
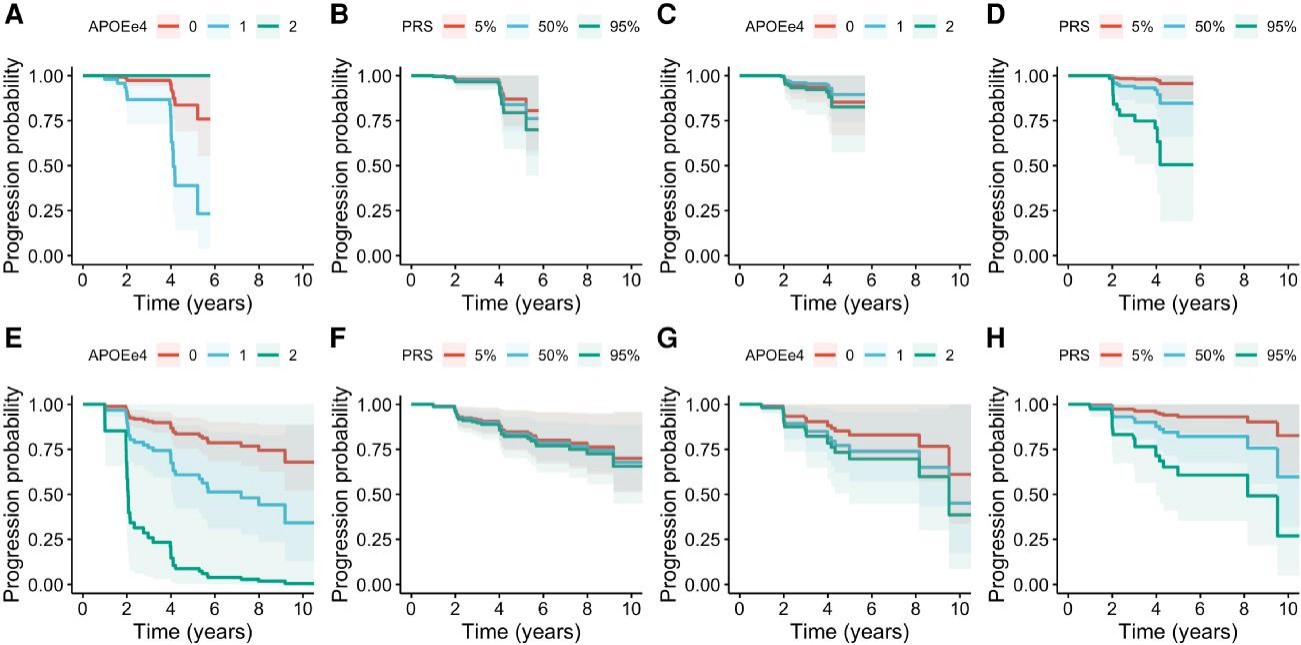
**Survival curves for PET-only and CSF-only analyses**. The *top row* (**A**–**D**) is based on results from AT(N) definitions based exclusively on PET biomarkers (amyloid and tau), with a cut-off of 1.45 for tau PET. The *bottom row* (**E**–**H**) relied on CSF biomarkers (ABETA42 and pTAU), with a cut-off of 26 for pTau. The *two left columns* (**A**, **B**, **E** and **F**) display the progression probability from A−T− to A+T− stratified by APOE-e4 genotype and PRS quantile (5%, 50% and 95%). The *two right columns* (**C**, **D**, **G** and **H**) display the progression probability from A+T− to A+T+.

The main analysis combined different biomarker sources to maximize the available data and the observation time for the conversion analysis. Relying on PET biomarkers alone, only 7.5% of visits (1237 of 16 401) had both biomarkers, and it resulted in shorter observation times for A−T− to A+T− conversion [3.62 (SD = 1.07) years] and A+T− to A+T+ conversion [2.94 (SD = 1.13) years] compared with the main analysis. Relying solely on CSF biomarkers, 19.2% of visits (3155 of 16 401) had both biomarkers. Overall, this led to a shorter observation time with respect to the main analysis for A−T− to A+T− conversion [3.94 (SD = 2.7) years] and A+T− to A+T+ conversions [3.6 (SD = 2.38) years]. Furthermore, relying on a single source of biomarkers led to reduced sample sizes (from ∼300 in the main analyses to 70–250 in the sensitivity analyses) and observed conversions (Supplementary Tables 5 and 6). Despite the reduced statistical power, these sensitivity analyses confirmed the pattern observed in the main analysis: APOE-e4 contributed mainly to the A−T− to A+T− conversion, whereas PRS contributed to the A+T− to A+T+ conversion (Fig. 3 and Supplementary Tables 5 and 6).

## Discussion

This longitudinal survival analysis demonstrated that APOE-e4 plays an important role for the progression from A−T− to A+T−, but *APOE* is of only marginal importance in A+T− participants who progress to A+T+. Conversely, polygenic risk for Alzheimer’s disease exhibited the inverse pattern: there was no contribution to the progression from A−T− to A+T−, but a significant contribution to faster progression from A+T− to A+T+. This held true for an alternative PRS defined using a different genetic study and involving a larger number of genetic loci. Notably, when assessing covariates, a differential effect of years of education was observed: higher education had no effect (Fig. 1) or was marginally harmful (Supplementary Fig. 2) for the progression from A−T− to A+T− but was protective for the conversion from A+T− to A+T+ (Fig. 2). The bisection of the genetic risk by disease stage was largely independent of the applied biomarker cut-offs. Moreover, relying on only a single source of biomarkers for defining stage and conversion confirmed the findings of the main analysis despite reduced sample size and observation time.

The finding of a stronger effect of APOE-e4 earlier in the disease progress might explain the observation of stronger genetic effect of APOE-e4 on Alzheimer’s disease in the group of 60- to 80-year-old people compared with people ≥80 years old.^57-59^ Given that amyloid deposition occurs 10+ years before other Alzheimer’s disease processes^2^ and APOE-e4 is the strongest common genetic risk factor for amyloid deposition, it would be expected for APOE-e4 to exert its maximum effect in younger people. Still, *APOE* remains the strongest risk factor in individuals ≥80 years old.^58^ Thus, the age-dependent heterogeneity of *APOE* is likely to be compounded by a survivor bias: individuals with a very late onset despite carrying APOE-e4 might harbour protective variants,^60^ such as KLOTHO-VS, where a protective effect on amyloid deposition and Alzheimer’s disease was observed in only 60- to 80-year-olds, but not in the 80+ group.^57,61^

The findings from this longitudinal analysis are also in line with previous reports in the ADNI cohort of *APOE* and polygenic risk on amyloid and tau. For instance, cross-sectional amyloid biomarkers in the CSF and in the brain were mainly driven by *APOE*, whereas cross-sectional CSF tTau and pTau measurements were associated with PRS beyond the *APOE* locus.^32^ Moreover, *APOE* was found to predict amyloid status, whereas polygenic risk for Alzheimer’s disease improved predictions of diagnosis and of clinical progression from mild cognitive impairment to Alzheimer’s disease above *APOE* alone.^35^ These observations extend to plasma markers of tau pathology: PRSs (that excluded the *APOE* region) were found to be associated only with plasma p-tau181 in A+ participants.^62^ This association between polygenic risk (beyond *APOE*) and CSF tau biomarkers rather than with amyloid and neurodegeneration was also observed outside of the ADNI study.^63^ Polygenic risk (beyond *APOE*) was associated with non-amyloid endophenotypes in a large cohort of people with mild cognitive impairment. This suggests that these variants are more closely linked with neuronal degeneration than with Alzheimer’s disease-related amyloid pathology.^64^ All these previous studies made the connection between existing amyloid pathology and correlations between polygenic risk and tau pathology using cross-sectional study designs. In a recent longitudinal study of tau PET in the ADNI cohort, higher polygenic risk for Alzheimer’s disease was associated with accelerated increase in tau signal in the brain, and this effect was modulated by amyloid pathology: A+ participants showed a stronger effect of PRS on tau accumulation.^65^ Our longitudinal analysis, which combined CSF and PET data to maximize the sample size, confirms these observations and indicates that polygenic Alzheimer’s disease risk (outside the *APOE* region) contributes to tau pathology in A+ participants but has no meaningful contribution in A− participants. The genetic architecture of Alzheimer’s disease, as captured by the PRS, involves multiple different pathways, mainly amyloid-β processing, tau, immunity and lipid processing.^18,21^ The PRS that we used covers three genes that have been associated with tau binding: *BIN1*, *CLU* and *PICALM*. *BIN1* mediates Alzheimer’s disease risk by modulating tau pathology,^66^ and *BIN1* risk variants increase tau PET (but not amyloid PET)^67^ in an amyloid-dependent fashion.^68^ Our observations of an amyloid-dependent effect of the PRS align with these earlier single-gene studies. Although there are currently no mechanistic analyses that explain the amyloid-dependent effect of *BIN1* on tau pathology, recent data from animal models in Alzheimer’s disease suggest a state-dependent effect of genetic risk factors related to microglia: deletion of *Trem2* in mouse models exacerbated tau accumulation and spreading, leading to brain atrophy, but only in the presence of existing amyloid-β pathology.^69^ Along the same lines, physical contact between microglia and plaques in addition to a functioning *TREM2* gene are necessary for the appropriate microglial response to amyloid pathology.^70^ Thus, defects in *TREM2* can contribute to neurodegeneration only once amyloid pathology has been established. Consequently, other genes contributing to the PRS might also exert their effect in an amyloid-dependent fashion. Our longitudinal analysis presented here is the first to support such a state-dependent genetic risk model in humans, and further fine-grained examination of how the pathways involved in the PRS contribute to sequential disease progression are needed.

The partition of Alzheimer’s disease genetic risk into *APOE*-related and polygenic risk beyond *APOE* is a simplification in this analysis. Recent works have linked established Alzheimer’s disease risk loci outside *APOE*, such as *CR1*, to amyloid biomarker levels.^23^ Conversely, studies of biomarker levels of tau repeatedly highlight the *APOE* locus.^23,24^ However, if being A+ were a prerequisite to exhibit pathological accumulation of tau, then the strong genetic association with *APOE* in these GWASs would merely reflect the necessary condition rather than a genuine direct molecular process that affects tau levels. The strong dependence of tau levels on established amyloid pathology is supported by mediation analyses in recent cross-sectional^63^ and longitudinal^65^ studies. Moreover, the known Alzheimer’s disease genetic risk variants are contributing differently to molecular pathways,^18,21,22^ where each pathway in turn will exercise differential effects on the Alzheimer’s disease biomarkers, including markers for vascular pathology.^37^ Therefore, a pathway PRS comprising only genes associated with the regulation of the amyloid precursor protein catabolic process (e.g. Gene Ontology term GO:1902991) might contribute significantly to the conversion from A−T− to A+T−. Likewise, a pathway PRS using only genes known to bind the tau protein (GO:0048156) might exhibit an even stronger association with A+T− to A+T+ conversion than the general PRS used here.

In addition to the cascading effect of genetic risk in Alzheimer’s disease, we also observed a stage-dependent effect of non-genetic risk factors. Education has been shown to have a protective effect against dementia^71^: here, we show that higher rates of education do not influence transition to amyloid positivity but do slow progression from A+T− to A+T+. Consequently, other non-genetic risk factors might show a similarly state-dependent effect on the pathological pathway from A−T− to A+T+ and further neurodegeneration.

The study has several limitations. First, the present analysis was limited to two biomarkers in Alzheimer’s disease: amyloid-β and tau. It would be desirable to include neurodegeneration (N; of the ATN framework) or, potentially, more fine-grade staging from advanced data-driven disease-progression modelling.^7^ However, at this point adding further stages would reduce the available sample size. Second, we partitioned the genetic risk in Alzheimer’s disease into two components: *APOE* and other top variants combined into a single polygenic risk score. Further work should explore a more fine-grained partition of the polygenic risk into individual SNPs or into pathway PRS.^37^ Third, the study population was of central European ancestry; therefore, it is unclear whether the findings would generalize to other genetic backgrounds. Finally, although the ADNI cohort is a large cohort, the number of subjects who were eligible for our analysis was reduced owing to the requirement for concordant and longitudinal recordings of multiple biomarkers in addition to genetics. The available sample size might have limited statistical power to render the estimated hazards ratios significant in some settings. However, the two conversion analyses were based on similar sample sizes (∼300 participants), thus allowing us to make a relative comparison between the genetic effect (of *APOE* or PRS) under two different biomarker-defined disease stages. Moreover, uncertainty of the estimated effects might also be increased owing to disease heterogeneity in Alzheimer’s disease,^72-75^ which is likely to be underpinned by differences in genetic architecture. Thus, analyses in further large longitudinal cohorts are required to confirm the observation of stage-dependent genetic vulnerability in Alzheimer’s disease and to uncover more fine-grained associations with Alzheimer’s disease subtypes.

## Conclusion

In this work we demonstrated, in a simplified setting, that genetic risk for late-onset Alzheimer’s disease unfolds in a disease stage-dependent fashion. A better understanding of the interplay between disease stage and genetic risk can lead to a better understanding of the molecular processes leading to Alzheimer’s disease, in addition to opening therapeutic windows for targeted interventions and personalized approaches to dementia prevention.

## Supplementary Material

awae176_Supplementary_Data

## Data Availability

Data used in the preparation of this article were obtained from the ADNI database (http://adni.loni.usc.edu) and are freely available after registration. Analysis scripts are available at: https://github.com/andrealtmann/cascading_genetic_risk.
