# Supplementary material for: Towards cascading genetic risk in Alzheimer’s disease
Source: Brain. 2024 May 31;147(8):2680–90. doi: 10.1093/brain/awae176 (PMC11292901; doi:10.1093/brain/awae176)
Supplement: awae176_Supplementary_Data [file awae176_supplementary_data.pdf]

Towards cascading genetic risk in Alzheimer's disease

Andre Altmann[1], Leon M Aksman[2], Neil P Oxtoby[1], Alexandra Young[1], ADNI*, Daniel C Alexander[1], Frederik Barkhof[1,3,4], Maryam Shoai[3,5], John Hardy[3,5], Jonathan M Schott[5,6]

1 UCL Centre for Medical Image Computing, Department of Medical Physics and Biomedical Engineering & Department of Computer Science, University College London, UK

2 Stevens Neuroimaging and Informatics Institute, Keck School of Medicine, University of Southern California, Los Angeles, CA 90033, USA

3 UCL Queen Square Institute of Neurology, University College London, UK

4 Department of Radiology and Nuclear Medicine, Amsterdam University Medical Center, The Netherlands

5 UK Dementia Research Institute, University College London, UK

6 Dementia Research Centre, UCL Queen Square Institute of Neurology, University College London, UK

Corresponding author:

Dr. Andre Altmann

90 High Holborn, 1st Floor

London, WC1V 6LJ

United Kingdom

Email: a.altmann@ucl.ac.uk

* Data used in preparation of this article were obtained from the Alzheimer's Disease Neuroimaging Initiative (ADNI) database (adni.loni.usc.edu). As such, the investigators within the ADNI contributed to the design and implementation of ADNI and/or provided data but did not participate in the analysis or the writing of this report. A complete list of ADNI investigators can be found at: http://adni.loni.usc.edu/wpcontent/uploads/how_to_apply/ADNI_Acknowledgement_List.pdf .

Supplementary Material

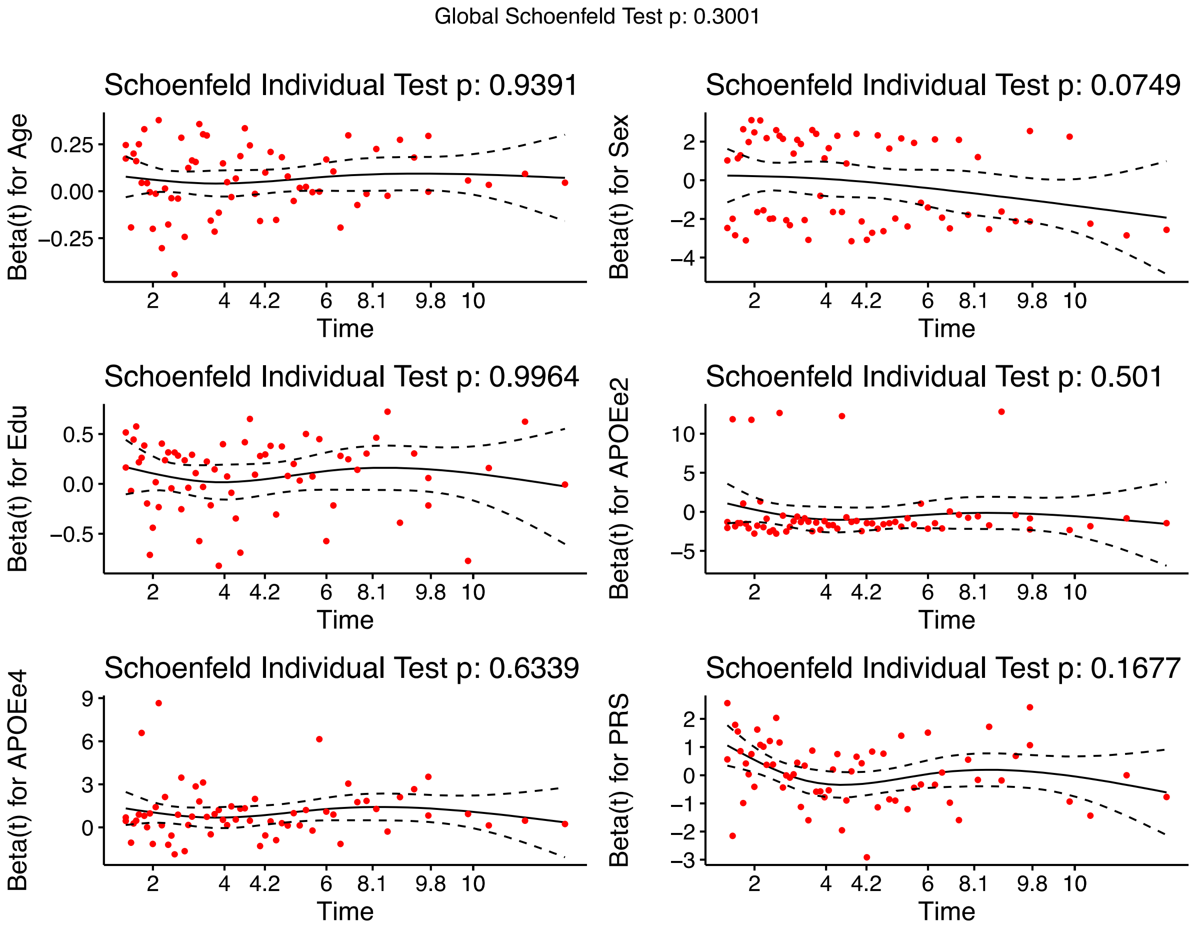

Supplementary Figure 1: Testing the Cox Proportional Hazards assumption for the Amyloid model. Each panel depicts the scaled Schoenfeld residuals for one covariate. The title of each panel states the p-value of the individual Schoenfeld tests. The title of the plot states the p-value of the global Schoenfeld test. None of the p-values are significant (i.e., $P<0.05$), therefore the Cox Proportional Hazards assumption holds.

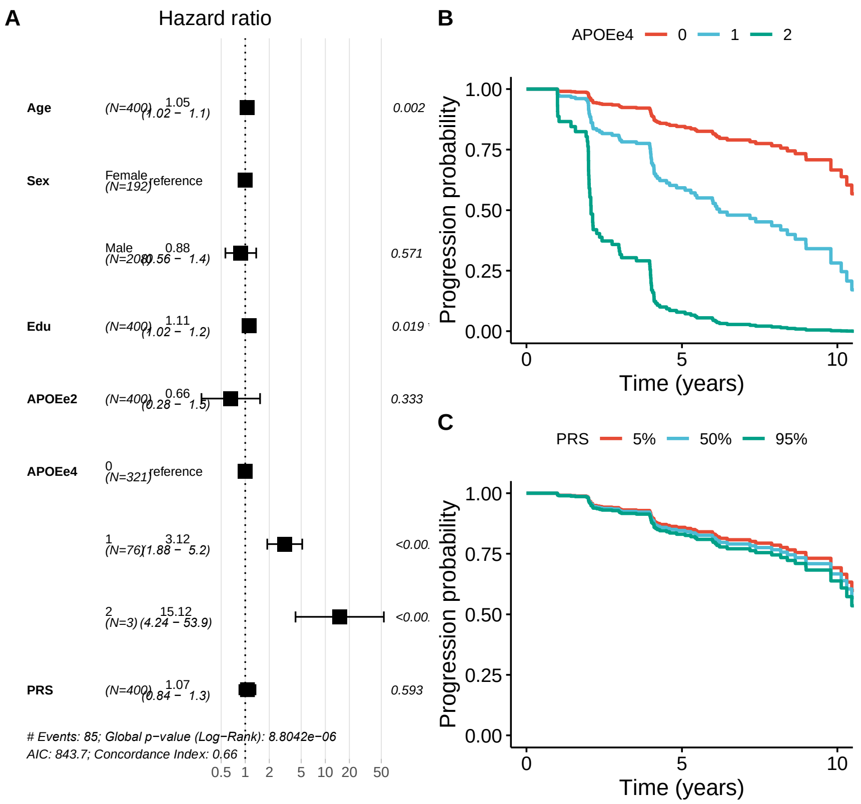

Supplementary Figure 2: Hazard ratios for the conversion from A-T- to A+. (A) Forest plot depicting the Hazards Ratios (HR) for all covariates in the model. (B) Estimated survival curves stratified by APOEe4 genotype. (C) Estimated survival curves stratified by PRS percentile (5%, 50%, 95%). Edu = Years of Educations; APOEe2 = number of APOE2 alleles, APOEe4 = number of APOE4 alleles; PRS = polygenic risk score, scaled to zero mean and unit standard deviation.

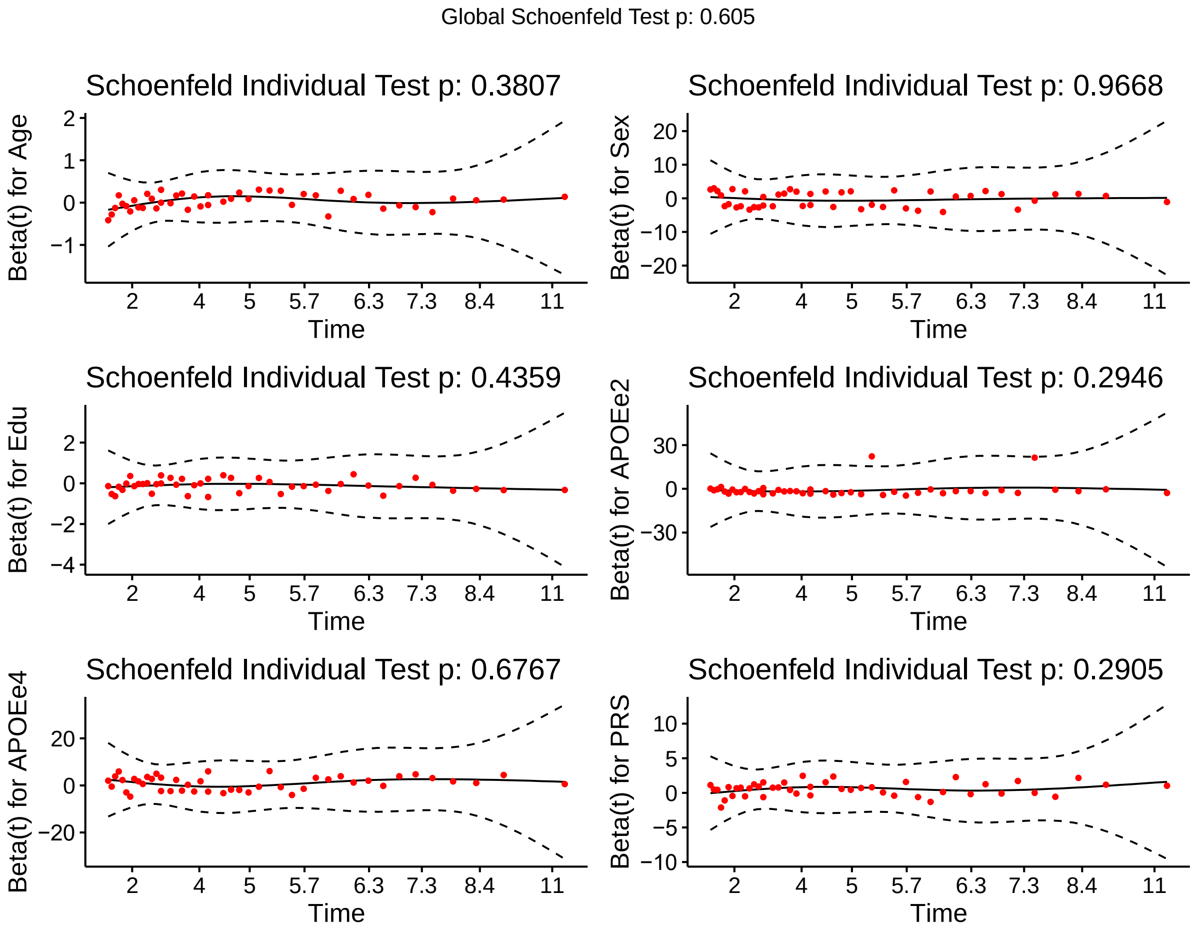

Supplementary Figure 3: Testing the Cox Proportional Hazards assumption for the Tau model. Each panel depicts the scaled Schoenfeld residuals for one covariate. The title of each panel states the p-value of the individual Schoenfeld tests. The title of the plot states the p-value of the global Schoenfeld test. None of the p-values are significant (i.e., P<0.05), therefore the Cox Proportional Hazards assumption holds.

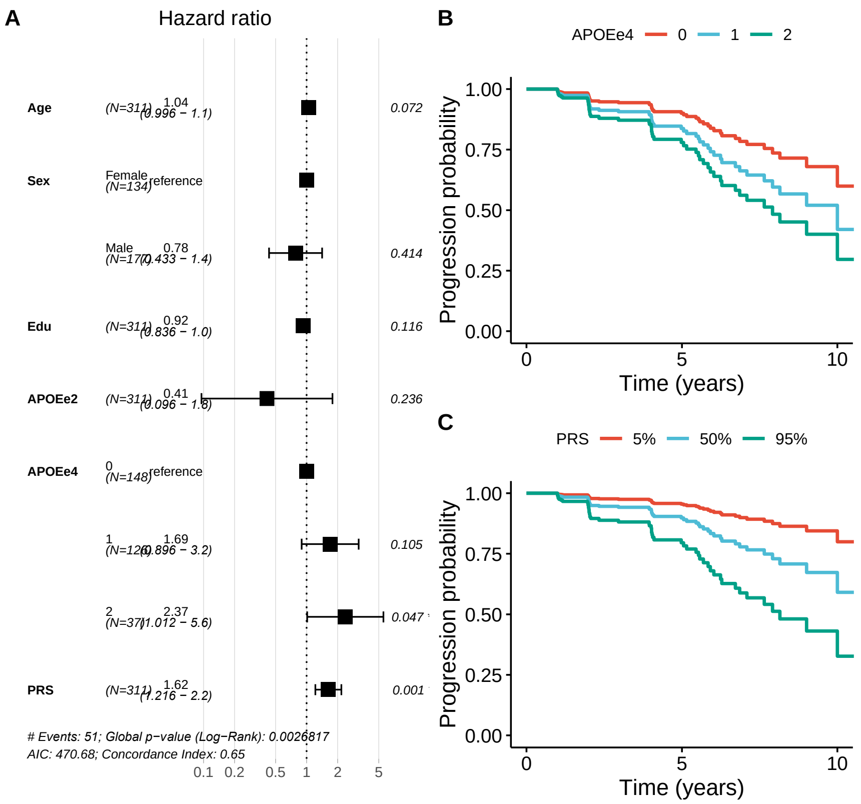

Supplementary Figure 4: Hazard ratios for the conversion from A+T- to T+. (A) Forest plot depicting the Hazards Ratios (HR) for all covariates in the model. (B) Estimated survival curves stratified by APOEe4 genotype. (C) Estimated survival curves stratified by PRS percentile (5%, 50%, 95%). Edu = Years of Educations; APOEe2 = number of APOE2 alleles, APOEe4 = number of APOE4 alleles; PRS = polygenic risk score, scaled to zero mean and unit standard deviation.

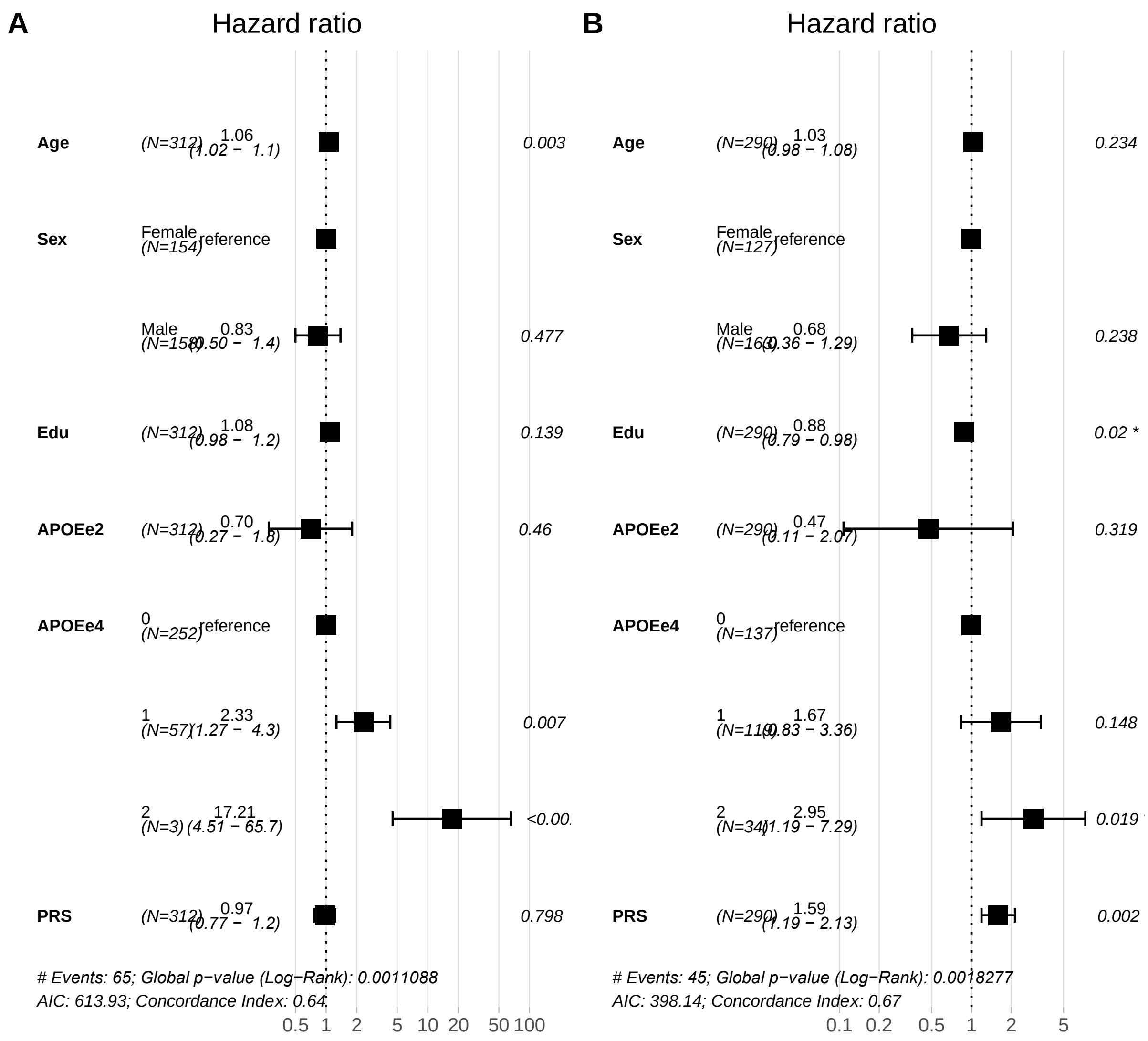

Supplementary Figure 5: Hazard ratios for the PRS with 77 SNPs based on Bellenguez *et al.* (2022). (A) Forest plot depicting the Hazards Ratios (HR) for all covariates in the model for the A-T- to A+T- conversion. (B) Forest plot depicting the Hazards Ratios (HR) for all covariates in the model for the A+T- to A+T+ conversion. Edu = Years of Educations; APOEe2 = number of APOE2 alleles, APOEe4 = number of APOE4 alleles; PRS = polygenic risk score, scaled to zero mean and unit standard deviation.

Supplementary Table 1: Genetic variants contributing to the Polygenic Risk Score.

Chr	Position	SNP	A1	A2	Gene	MAF	Weight	P-value
1	207750568	rs679515	T	C	*CR1*	0.1778	0.1508	1.56E-16
2	127889932	rs6710467	A	G	*BIN1*	0.1706	0.133	8.96E-12
2	127892810	rs6733839	T	C	*BIN1*	0.3879	0.1693	4.02E-28
8	27219987	rs73223431	T	C	*PTK2B*	0.3578	0.0936	8.34E-10
8	27468503	rs867230	C	A	*CLU*	0.3958	-0.1333	3.49E-17
11	47380340	rs3740688	G	T	*SPI1*	0.4331	-0.0935	9.70E-11
11	47663049	rs10838738	G	A	*MTCH2*	0.3348	-0.0871	5.99E-09
11	47915299	rs34467936	G	A	*PTPRJ*	0.3372	-0.0905	7.89E-09
11	60021948	rs1582763	A	G	*MS4A4A*	0.3516	-0.1232	1.19E-16
11	85868640	rs3851179	T	C	*PICALM*	0.3443	-0.1198	5.81E-16
14	92938855	rs12590654	A	G	*SLC24A4*	0.3402	-0.0906	8.73E-09
19	1047078	rs4147910	G	A	*ABCA7*	0.1077	0.1488	9.27E-09
19	1050874	rs12151021	A	G	*ABCA7*	0.3371	0.1071	2.56E-10

Chr: Chromosome; Position: position of the SNP in hg19; A1: effect allele; A2: reference allele; Gene: closest gene; MAF: minor allele frequency in the joint ADNI data (*n*=2001); Weight: beta coefficient from the genome-wide association study (GWAS); P-value: p-value from the stage 1 GWAS by Kunkle et al. (2019).

Supplementary Table 2: Genetic variants contributing to the alternative Polygenic Risk Score.

Chr	Position	SNP	A1	A2	*Gene*	EAF	Weight	P-value
1	161185602	rs4575098	A	G	*ADAMTS4*	0.2404	0.0539	1.33E-08
1	161217875	rs2070902	T	C	*FCER1G*	0.2539	-0.0537	1.05E-08
1	207221638	rs6540874	T	C	*C4BPA*	0.7938	-0.0706	1.70E-12
1	207518704	rs6656401	A	G	*CR1*	0.1881	0.1253	2.84E-33
2	127042739	rs56049505	T	G	*BIN1*	0.7987	-0.0618	1.14E-09
2	127072128	rs75836995	T	C	*BIN1*	0.7892	0.0816	6.92E-16
2	127072643	rs17014873	A	C	*BIN1*	0.9293	-0.1166	2.36E-13
2	127130409	rs12989701	A	C	*BIN1*	0.1522	0.0802	8.02E-13
2	127133508	rs34546266	A	G	*BIN1*	0.848	-0.0762	2.64E-11
2	127135234	rs6733839	T	C	*BIN1*	0.3891	0.1686	6.48E-90
2	233117495	rs7421448	T	C	*INPP5D*	0.0968	-0.1091	1.01E-13
2	233160866	rs28459768	A	C	*INPP5D*	0.5186	-0.0608	1.31E-13
4	993555	rs3822030	T	G	*IDUA*	0.5712	0.0514	5.04E-10
4	11023507	rs6846529	T	C	-	0.7174	-0.0673	1.25E-13
5	87002714	rs62375397	T	C	-	0.2104	0.0746	8.58E-14
5	87111682	rs2624182	A	G	-	0.7405	0.0517	3.94E-08
5	180201150	rs113706587	A	G	*RASGEF1C*	0.1103	0.0925	3.38E-12
6	32373621	rs9391858	A	G	*TSBP1*	0.8475	-0.068	2.79E-09
6	32408571	rs3763312	A	G	*BTNL2*	0.2036	-0.0708	5.88E-12
6	32713500	rs2858331	A	G	*HLA-DQA2*	0.5891	-0.0544	8.76E-11
6	32714360	rs3957148	A	G	*HLA-DQA2*	0.8996	0.1066	7.93E-15
6	41192063	rs9394766	A	G	*TREML2*	0.7013	0.0616	3.27E-12
6	41272084	rs4714447	T	C	*TREM1*	0.3449	0.0578	1.73E-11
6	47543755	rs9349413	A	G	*CD2AP*	0.735	-0.0649	1.61E-12
6	114361563	rs976271	A	G	*HS3ST5*	0.3613	0.0467	3.24E-08
7	28135367	rs67250450	T	C	*JAZF1*	0.7875	0.0559	2.02E-08
7	54881563	rs74504435	A	G	-	0.9065	0.0842	2.05E-09
7	100045122	rs4424195	A	G	*ZKSCAN1*	0.7209	0.057	3.21E-10
7	100176704	rs12705074	A	G	*GPC2*	0.1121	0.0731	1.80E-08
7	100334426	rs7384878	T	C	*PMS2P1*	0.69	0.0775	2.13E-18
7	100489836	rs13235951	T	C	*NYAP1*	0.1266	0.0751	9.56E-10
7	100592493	rs2734895	T	C	*FBXO24*	0.3128	-0.0586	6.58E-11
7	143413669	rs11771145	A	G	*EPHA1*	0.3476	-0.0604	1.29E-12
8	27362470	rs73223431	T	C	*PTK2B*	0.3694	0.0656	5.34E-15
8	27545260	rs7341557	A	G	*EPHX2*	0.1011	-0.0986	8.78E-13
8	27562086	rs11780834	A	G	*GULOP*	0.8236	-0.0626	3.42E-09
8	27610986	rs867230	A	C	*CLU*	0.6031	0.1009	1.50E-33
8	94988691	rs4734295	A	G	*NDUFAF6*	0.5394	-0.0487	1.98E-09
8	100663356	rs1693551	T	C	*SNX31*	0.5328	-0.0459	1.79E-08
10	11450869	rs11257101	T	C	*USP6NL*	0.6898	0.0481	4.69E-08
10	11676714	rs7912495	A	G	*ECHDC3*	0.5381	-0.0572	2.87E-12
10	60025170	rs7068231	T	G	*ANK3*	0.4026	-0.0487	6.79E-09
10	80494228	rs6586028	T	C	*TSPAN14*	0.8036	0.0791	1.33E-14
11	47389337	rs10838702	T	G	*SLC39A13*	0.4008	0.0565	7.84E-12
11	47759202	rs7927445	T	G	*FNBP4*	0.3105	0.0498	1.29E-08
11	60254475	rs1582763	A	G	*MS4A4A*	0.371	-0.086	1.65E-24
11	86095220	rs568755	A	G	*PICALM*	0.6909	-0.05	1.23E-08
11	86152038	rs56157503	A	C	*PICALM*	0.8768	-0.0882	7.69E-13
11	86156833	rs10792832	A	G	*PICALM*	0.3578	-0.1056	6.33E-36
11	121487553	rs60228070	T	C	*SORL1*	0.8997	-0.0756	2.70E-08

11	121566862	rs1784920	A	G	*SORL1*	0.1215	-0.0827	1.35E-10
14	39418472	rs74745468	A	G	*FBXO33*	0.0875	0.0822	1.40E-08
14	52924962	rs17125924	A	G	*FERMT2*	0.9108	-0.0881	5.82E-10
14	92472511	rs12590654	A	G	*SLC24A4*	0.3279	-0.0693	2.08E-15
15	49901356	rs2009833	A	G	*ATP8B4*	0.3683	-0.0478	3.11E-08
15	58753575	rs593742	A	G	*ADAM10*	0.7052	0.061	1.04E-11
15	63312881	rs16946801	T	C	*APH1B*	0.6204	0.0622	1.15E-13
16	30022312	rs12325539	T	C	*DOC2A*	0.6189	0.0567	1.25E-11
16	31143037	rs78924645	A	G	*PRSS36*	0.2795	-0.0565	7.71E-10
16	81739604	rs12444183	A	G	*PLCG2*	0.3872	-0.0591	2.21E-12
16	81942226	rs4485362	T	G	*PLCG2*	0.5396	0.0463	2.00E-08
16	90103687	rs56407236	A	G	*FAM157C*	0.0693	0.1097	1.28E-11
17	5220566	rs113762960	A	G	*SCIMP*	0.129	0.0864	7.78E-13
17	44352876	rs5848	T	C	*GRN*	0.2886	0.0646	1.76E-12
17	45846317	rs12185268	A	G	*SPPL2C*[a]	0.774	0.0536	3.85E-08
17	46107462	rs4510068	T	G	*KANSL1*[a]	0.3938	-0.0546	1.37E-10
17	46720553	rs199456	T	C	*NSF*[a]	0.2123	-0.0593	2.57E-09
17	58363181	rs2680700	T	G	*RNF43*	0.3755	-0.0482	9.90E-09
17	63476980	rs4292	T	C	*ACE*	0.617	0.0688	3.47E-16
19	1032690	rs1141534	T	G	*CNN2*	0.204	0.073	3.65E-11
19	1047079	rs4147910	A	G	*ABCA7*	0.8995	-0.0977	1.47E-11
19	1050875	rs12151021	A	G	*ABCA7*	0.3357	0.1055	4.09E-30
19	1085456	rs72975514	T	C	*ARHGAP45*	0.7924	0.06	4.60E-08
19	1833339	rs732310	T	G	*REXO1*	0.5158	-0.05	1.33E-08
20	56423488	rs6014724	A	G	*CASS4*	0.9102	0.1176	4.84E-16
21	26101558	rs2154481	T	C	*APP*	0.5236	0.05	1.02E-09
21	26775872	rs2830489	T	C	*ADAMTS1*	0.2809	-0.0547	1.72E-09

Chr: Chromosome; Position: position of the SNP in hg38; A1: effect allele; A2: reference allele; Gene: closest protein-coding gene within 100kb; EAF: effect allele frequency; Weight: beta coefficient from the genome-wide association study (GWAS); P-value: p-value from the stage 1 GWAS by Bellenguez *et al.* (2022). [a]: *MAPT* locus

Supplementary Table 3: Demographics (relaxed conversion criterion).

	A-T-			A+T-		
	**Total**	**Stable**	**Converter**	**Total**	**Stable**	**Converter**
N	400	315	85	311	260	51
Age, y (sd)	71.0 (6.9)	70.8 (6.7)	72.1 (7.1)	73.2 (7.0)	73.1 (7.0)	73.5 (7.0)
Sex (%female)	48.0	47.6	49.4	43.1	42.7	45.1
DX (CN/MCI/AD)	229/166/5	174/137/4	55/29/1	131/161/19	112/131/17	19/30/2
Education, y (sd)	16.7 (2.4)	16.6 (2.5)	17.2 (2.2)	16.5 (2.6)	16.6 (2.6)	15.8 (2.4)
Years Follow-up, y (sd)	4.9 (3.0)	4.6 (2.8)	6.3 (3.4)	4.0 (2.7)	3.7 (2.6)	5.5 (2.4)
Time to event, y (sd)	n/a	n/a	4.2 (2.7)	n/a	n/a	4.0 (2.4)
APOE-e4 (0/1/2)	321/76/3	266/49/0	55/27/3	148/126/37	129/103/28	19/23/9
APOE-e2 (0/1/2)	347/52/1	268/46/1	79/6/0	291/20/0	242/18/0	49/2/0
PRS (sd)	0.013 (0.012)	0.013 (0.012)	0.013 (0.011)	0.014 (0.013)	0.013 (0.013)	0.019 (0.012)

DX = diagnosis (cognitively normal [CN]; mild cognitive impairment [MCI]; Alzheimer's Disease (AD); PRS = Polygenic Risk Score

Supplementary Table 4: Sensitivity analysis for different Tau PET cutoffs.

	Tau PET	CSF pTau	*n*	Events	APOE e4	APOE e4/4	PRS
A-T- to A+T-	1.31	32.30	309	62	0.02915643	4.06E-05	0.6499623
	1.34	32.95	310	64	0.01775002	3.55E-05	0.5021401
	1.37	33.57	311	65	0.00726623	2.20E-05	0.4358987
	1.39	34.13	311	65	0.00699722	2.02E-05	0.5160777
	**1.42**	**34.61**	**312**	**65**	**0.00639089**	**1.81E-05**	**0.4962599**
	1.45	34.82	314	67	0.00904438	1.41E-05	0.5737404
	1.48	35.07	315	67	0.00896343	1.39E-05	0.5734747
	1.51	35.29	315	67	0.00896343	1.39E-05	0.5734747
	1.54	35.53	316	67	0.00885485	1.38E-05	0.5735298
A+T- to A+T+	1.31	32.30	258	46	0.05796633	0.48549513	0.00204043
	1.34	32.95	266	44	0.10834803	0.2154368	0.00084655
	1.37	33.57	271	42	0.06722642	0.04442165	0.00097163
	1.39	34.13	280	43	0.07594692	0.09580913	0.00069806
	**1.42**	**34.61**	**290**	**45**	**0.11930622**	**0.03939911**	**0.0005569**
	1.45	34.82	290	43	0.18026366	0.02915812	0.00089779
	1.48	35.07	291	39	0.55440604	0.02044481	0.0076896
	1.51	35.29	293	41	0.30800169	0.02089209	0.0048998
	1.54	35.53	296	39	0.37466685	0.02998641	0.00162742

Tau PET: Tau PET threshold based on 1.0 to 3.0 standard deviations (in steps of 0.25) above the mean in cognitively normal participants. CSF pTau: pTau threshold that maximized the Youden's index for the corresponding Tau PET cutoff. *n*: sample size; Events: number of converters; APOE e4: p-value for APOEe4 heterozygotes; APOE e4/4: p-value for APOEe4 homozygotes; PRS: p-value for the Polygenic Risk Score. Bold font indicates the settings used for the main analysis (i.e., Tau PET cutoff at a z-score of 2.0).

Supplementary Table 5: Sensitivity analysis using only Tau PET.

	Tau PET	*n*	Events	APOE e4	APOE e4/4	PRS
A-T- to A+T-	1.31	108	15	0.00256332	0.998414	0.5466185
	1.34	110	17	0.00617315	0.998169	0.375093
	1.37	112	18	0.00194	0.9983591	0.3558922
	1.39	113	18	0.0019078	0.9983644	0.3514293
	1.42	113	18	0.0019078	0.9983644	0.3514293
	**1.45**	**113**	**19**	**0.00276536**	**0.9982344**	**0.5255481**
	1.48	113	19	0.00276536	0.9982344	0.5255481
	1.51	113	19	0.00276536	0.9982344	0.5255481
	1.54	113	19	0.00276536	0.9982344	0.5255481
A+T- to A+T+	1.31	70	9	0.4401025	0.9988313	0.1140501
	1.34	73	8	0.3714046	0.9987921	0.21299683
	1.37	78	9	0.2152745	0.2792531	0.15481342
	1.39	80	7	0.5417669	0.2673798	0.02184563
	1.42	90	13	0.4710315	0.6737454	0.01884522
	**1.45**	**91**	**13**	**0.6017861**	**0.8375521**	**0.01031391**
	1.48	91	10	0.2393688	0.4005217	0.04235292
	1.51	92	11	0.3619229	0.3545669	0.03555231
	1.54	94	12	0.14025	0.1759473	0.00334521

Tau PET: Tau PET threshold based on 1.0 to 3.0 standard deviations (in steps of 0.25) above the mean in cognitively normal participants. *n*: sample size; Events: number of converters; APOE e4: p-value for APOEe4 heterozygotes; APOE e4/4: p-value for APOEe4 homozygotes; PRS: p-value for the Polygenic Risk Score. Bold font indicates the settings presented in the main analysis (i.e., Tau PET cutoff at a z-score of 1.0).

Supplementary Table 6: Sensitivity analysis using only CSF pTau.

	CSF pTau	*n*	Events	APOE e4	APOE e4/4	PRS
A-T- to A+T-	22	218	28	0.07085586	1.39E-01	0.9051517
	23	229	30	0.14716628	1.96E-01	0.9600861
	24	236	32	0.06568113	1.86E-01	0.6503611
	25	246	35	0.00771617	1.63E-01	0.5129081
	**26**	**257**	**38**	**0.01069569**	**2.81E-04**	**0.7715454**
	27	263	42	0.0045226	2.65E-04	0.9991425
	28	269	43	0.00171823	1.66E-04	0.9664246
	29	276	44	0.00179873	1.40E-04	0.7945879
	30	279	45	0.000537	1.06E-04	0.6305412
	31	286	47	0.00106118	7.44E-05	0.4812926
A+T- to A+T+	22	91	24	0.01928131	0.00271671	0.41947419
	23	99	21	0.13332607	0.05169251	0.51483344
	24	109	20	0.17550508	0.15774646	0.34919713
	25	115	22	0.20414182	0.58489759	0.03751601
	**26**	**121**	**21**	**0.34477282**	**0.38195035**	**0.0102725**
	27	130	17	0.91675502	0.66832828	0.00761942
	28	136	14	0.76128045	0.77591764	0.07535193
	29	145	12	0.42916222	0.93156112	0.02431801
	30	148	12	0.29036457	0.92810651	0.08045729
	31	154	11	0.6390905	0.61806754	0.04685604

CSF pTau: pTau threshold ranging from 22 to 31 pg/ml. *n*: sample size; Events: number of converters; APOE e4: p-value for APOEe4 heterozygotes; APOE e4/4: p-value for APOEe4 homozygotes; PRS: p-value for the Polygenic Risk Score.
